# Zein Protein Nanoparticles: Fabrication and Rheology of Their Dispersions in Polysaccharides

**DOI:** 10.3390/foods15193529

**Published:** 2026-10-02

**Authors:** Siyi Li, Rajinder Pal

**Affiliations:** Department of Chemical Engineering, University of Waterloo, Waterloo, ON N2L 3G1, Canada; s998li@uwaterloo.ca

**Keywords:** zein, zein nanoparticles, polysaccharide, xanthan gum, sodium carboxymethyl cellulose, rheology, non-Newtonian, viscosity, consistency, surface tension

## Abstract

Zein protein nanoparticles are fabricated in two anionic polysaccharides, sodium carboxymethyl cellulose (CMC) and xanthan gum (XG), using the anti-solvent method. The rheological behavior and surface-active properties of dispersions of zein nanoparticles in polysaccharides are investigated. Two sets of experiments are carried out. In one set, the zein concentration is fixed at 1 wt% and the concentrations of the polysaccharides varied from 0 to 1 wt% in increments of 0.2 wt%. In the second set of experiments, the zein concentration varies from 1 to 5 wt% at a fixed polysaccharide concentration of 0.5 wt%. The SEM images clearly show the formation of zein nanoparticles in polysaccharides. Polysaccharide solutions and zein nanoparticle-polysaccharide dispersions exhibit shear-thinning rheological behavior and follow the power-law model adequately. With the addition of zein, the consistency of zein nanoparticle–CMC dispersions increases, and they become more shear-thinning in comparison to pure CMC solutions. With the increase in zein concentration at a fixed CMC concentration, the consistency rises sharply up to 4 wt% zein concentration and then drops substantially, with a further increase in zein concentration. The addition of zein to XG solutions exhibits a more complex rheological behavior. The consistency index decreases with the addition of zein to XG solutions when XG concentration is less than 0.7 wt%. At higher XG concentrations, the consistency index increases with the addition of zein to XG solutions. With the increase in zein concentration at a fixed XG concentration, the consistency decreases substantially over the full range of zein concentration investigated. The surface tension of CMC solutions is strongly affected by the addition of zein nanoparticles. It decreases substantially with the addition of zein to CMC solutions. The surface tension behavior of zein nanoparticle-XG dispersions is like that of zein nanoparticle–CMC dispersions. The transitions in rheological and surface-active properties of zein nanoparticle-polysaccharide dispersions are explained in terms of microstructure of the dispersions.

## 1. Introduction

Zein, a hydrophobic prolamin protein derived from maize, has gained increasing attention in recent years as a biodegradable material for food and biopolymer applications, particularly in encapsulation and structured delivery systems [1,2]. Its amphiphilic molecular structure enables self-assembly into colloidal particles under selective solvent conditions, especially through anti-solvent precipitation. However, the high content of nonpolar amino acids in zein leads to poor water solubility and strong intermolecular hydrophobic interactions, resulting in aggregation and limited colloidal stability in aqueous environments [2,3]. These intrinsic limitations significantly restrict its broader application in food systems where stability and tunable rheological properties are critical.

To address these challenges, polysaccharides have been widely introduced as functional modifiers to improve the dispersion stability and rheological performance of zein-based systems. Sodium carboxymethyl cellulose (CMC) and xanthan gum (XG) are two representative anionic polysaccharides commonly used as thickening and stabilizing agents in food formulations. CMC is a flexible, water-soluble cellulose derivative whose rheological behavior is governed by chain entanglement and electrostatic repulsion, resulting in moderate but highly tunable viscosity [4,5]. In contrast, XG is a high-molecular-weight, semi-flexible polysaccharide with a rigid helical conformation and strong shear-thinning behavior, which allows it to generate high viscosity even at low concentrations [6,7]. Its charged side chains and molecular rigidity contribute to strong intermolecular interactions and network formation. Due to these structural differences, CMC and XG exhibit distinct interaction mechanisms with proteins, which may lead to fundamentally different macroscopic behaviors when combined with zein. Beyond their contrasting molecular architectures, CMC and XG were also selected for their established, food-grade functional roles: CMC is among the most widely used thickeners and stabilizers in dairy products, sauces, beverages, and baked goods, valued for its low cost and highly tunable viscosity, while XG is extensively used as a stabilizer in salad dressings, sauces, and gluten-free formulations owing to its pronounced shear-thinning behavior and stability across a wide range of processing conditions. Zein, in turn, is a GRAS, plant-derived protein increasingly explored as a natural stabilizer for Pickering emulsions and as a carrier for the encapsulation of lipophilic bioactive compounds. Bare zein nanoparticles, however, are highly hydrophobic and prone to aggregation in aqueous media; formulating them with CMC or XG is expected to provide electrostatic and/or steric stabilization, consistent with the known polyelectrolyte character of these polysaccharides, while imparting tunable, shear-thinning rheology. The combination of zein nanoparticles with CMC or XG is therefore directly relevant to the development of protein-fortified, plant-based food products requiring both colloidal stability and processing-friendly rheology, as well as to encapsulation systems for functional food and nutraceutical applications.

The incorporation of polysaccharides has emerged as an effective strategy to enhance dispersion stability and tailor functional properties of zein. Practically, this is mostly achieved utilizing the anti-solvent precipitation method. In this process, zein is first dissolved in aqueous ethanol and subsequently introduced into an aqueous polysaccharide solution, triggering rapid de-solvation and the self-assembly of zein into nanoparticles [1,8,9]. Importantly, the presence of polysaccharides during this precipitation phase triggers a ‘kinetic arrest’; they immediately adsorb onto the newly formed zein nuclei to provide robust steric or electrostatic stabilization, thereby locking the particle size at the nanoscale and preventing macroscopic aggregation [10,11].

The specific internal morphology of these nanocomplexes heavily depends on the structural properties of the continuous polysaccharide phase. For instance, zein–CMC complexes formed via this route typically generate a colloidal dispersion characterized by a partial core–shell structure, where flexible CMC chains adsorb onto zein surfaces and induce inter-particle bridging. In contrast, zein–xanthan gum (XG) complexes exhibit a distinct concentration-dependent microstructural evolution. At sufficient XG concentrations (≥0.8 wt%), a dense rigid network dominates the continuous phase, and the positively charged zein particles act as multifunctional junction points through strong electrostatic bridging, significantly enhancing the overall network connectivity [12,13]. Therefore, the anti-solvent method coupled with polysaccharide modification provides a highly controllable platform for developing structured biopolymer systems.

Despite these advances, important knowledge gaps remain. Most existing studies on zein–polysaccharide systems primarily focus on particle formation, interfacial behavior, or emulsion stabilization, while comparatively fewer studies systematically address the bulk rheological properties of these systems. For example, recent work on zein-based gels and composites have largely emphasized structural formation and stability rather than detailed viscosity evolution across concentration regimes [14,15,16]. Similarly, studies on zein–polysaccharide blends (e.g., with glucomannan or chitosan) report changes in microstructure and rheology but do not comprehensively investigate how viscosity evolves with varying polysaccharide concentration [17].

To bridge these knowledge gaps, the present study aims to systematically investigate the concentration-dependent rheological behavior of zein–polysaccharide composite systems fabricated via the anti-solvent precipitation method. Specifically, zein is combined with two representative but structurally distinct polysaccharides, sodium carboxymethyl cellulose (CMC) and xanthan gum (XG), to construct colloidal dispersions with tunable properties. By systematically varying both the type and concentration of the polysaccharides, the steady-shear flow behavior and viscosity evolution of the resulting systems are comprehensively evaluated. Special attention is given to elucidating how the intrinsic molecular characteristics of CMC and XG translate into the macroscopic flow behavior and phase stability of zein-based dispersions. Ultimately, this comparative analysis provides critical insights into the structure–property relationships of protein–polysaccharide complexes, offering valuable theoretical guidance for predicting their processing performance and optimizing their structural stability in advanced food and biopolymer applications.

The unique rheological and surface-active properties of the zein–polysaccharide dispersions investigated in this study have important implications for food formulation. The pronounced shear-thinning behavior and high consistency observed in these systems are highly desirable for texture modification in semi-solid foods, such as sauces, dips, and dressings, where high resting viscosity prevents phase separation and easy flow under shear facilitates processing and consumption. Furthermore, the significant reduction in surface tension upon the incorporation of zein nanoparticles highlight their potential utility as plant-based Pickering emulsifiers or foam stabilizers for products like egg-free mayonnaise or aerated desserts. Finally, while future structural characterization and specific encapsulation assays are required for validation, the hypothesized core–shell-type morphology of these nanocomplexes provides a promising structural basis for developing structured delivery systems to encapsulate hydrophobic bioactives in functional beverages.

## 2. Materials and Methods

### 2.1. Materials

The sodium carboxymethyl cellulose (CMC) used in this work was manufactured by Hercules Inc., Wilmington, DE, USA, under the trade name of Hercules cellulose gum. The approximate molecular weight of the polymer was $7\times 10^{5}$ with degree of substitution in the range of 0.65–0.90. Xanthan gum (XG) was manufactured by CP Kelco, Atlanta, GA, USA, under the trade name of Kelzan. The approximate molecular weight of the polymer was $2\times 10^{6}$. Zein was purchased from Sigma Aldrich Canada, Oakville, ON, Canada.

CMC is an anionic water-soluble cellulose derivative widely used as a thickening and stabilizing agent in food and pharmaceutical applications due to its excellent rheological and emulsifying properties. Xanthan gum (XG) is an anionic extracellular polysaccharide produced by bacterial fermentation, known for its high viscosity and strong shear-thinning behavior even at low concentrations. Zein, the major storage protein of corn, is a hydrophobic biopolymer that is commonly used for film formation and encapsulation purposes owing to its self-assembly behavior in aqueous ethanol systems.

### 2.2. Preparation of Zein Stock Solutions

Zein powder was dissolved in 90% (*w*/*w*) aqueous ethanol solution and homogenized for 1 h at room temperature to obtain a 10 wt% zein stock solution.

### 2.3. Preparation of Polysaccharide-Zein Composite Systems

**Figure 1:**
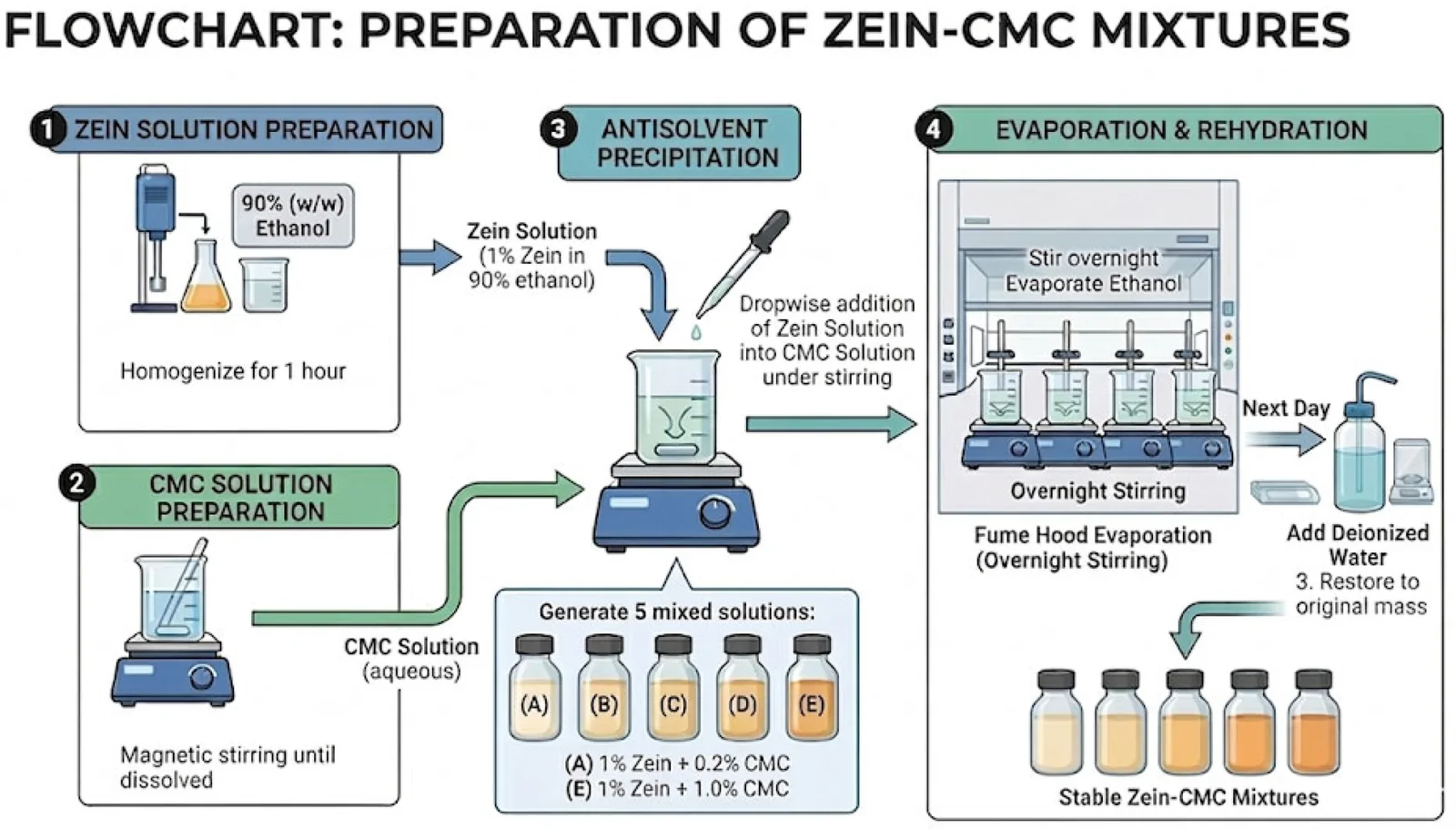
Schematic illustration of the preparation of zein–CMC composite dispersions via anti-solvent precipitation.

**Figure 2:**
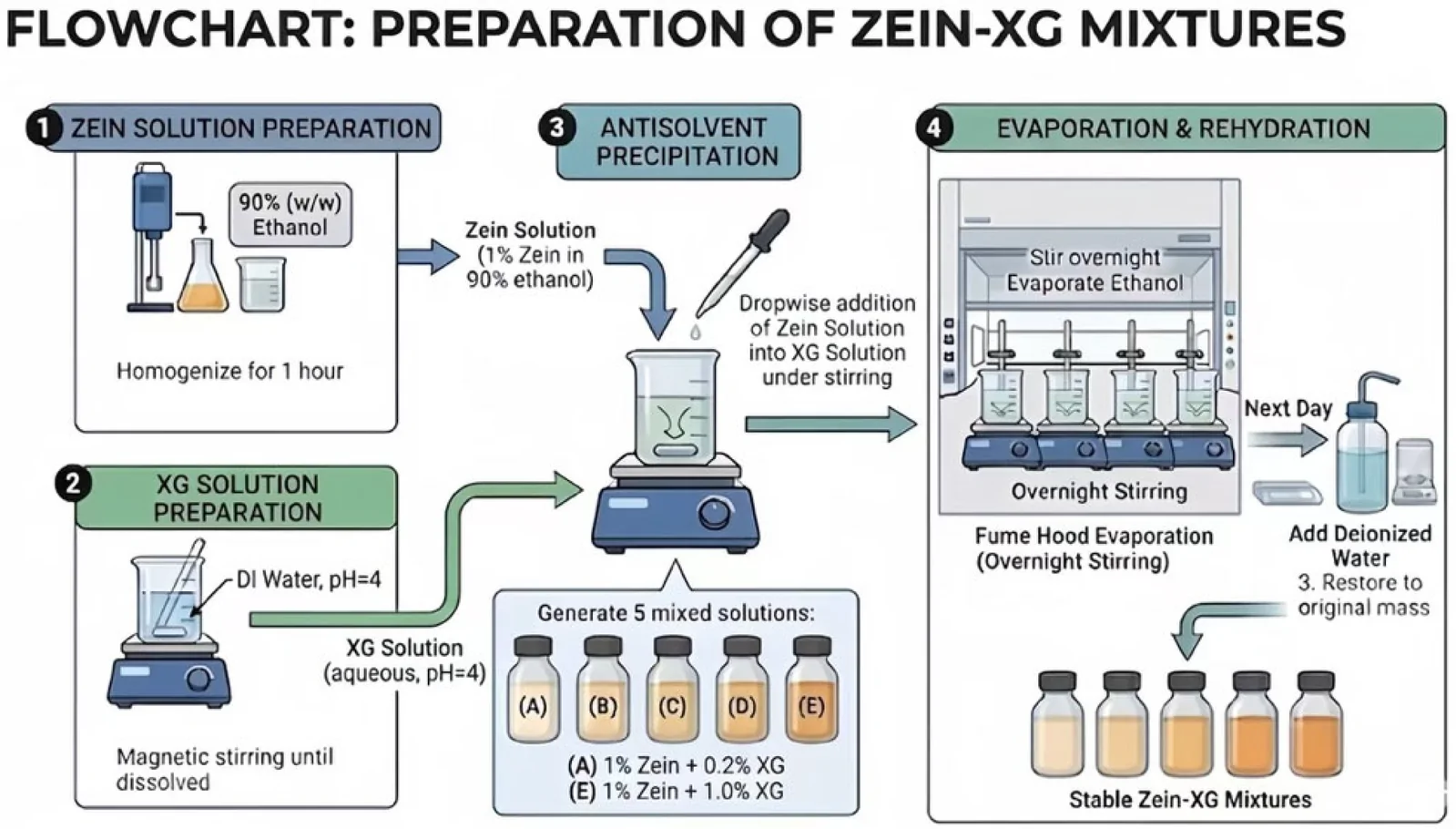
Schematic illustration of the preparation of zein–XG composite dispersions under acidic conditions (pH 4.0).

Polysaccharide-zein composite systems were fabricated utilizing an anti-solvent precipitation method. The overall preparation and solvent-evaporation processes are schematically summarized in Figure 1 and Figure 2. To systematically investigate the effects of biopolymer mixing ratios, two experimental series were designed. In all formulations, the total initial mass of the mixture was standardized to 400 g prior to solvent evaporation.

Series I: Effect of varying polysaccharide concentration. CMC or XG was dispersed in deionized water to prepare solutions with concentrations ranging from 0.2 to 1.0 wt%. For XG solutions, the pH was pre-adjusted to 4.0 using 0.1 M HCl to promote strong electrostatic interactions with the positively charged zein (pI ≈ 6.2) [18,19]. Subsequently, 40 g of the 10 wt% zein stock solution was added dropwise into the polysaccharide solutions under continuous magnetic stirring (1200 rpm), yielding a constant final zein concentration of 1 wt% [20,21]. No pH adjustment was applied to the CMC systems.

Series II: Effect of varying zein concentration. A constant polysaccharide (CMC or XG) concentration of 0.5 wt% was utilized as the continuous phase. Prior to mixing, the pH of the XG solutions was adjusted to 4.0, whereas the CMC solutions were used without any pH modification. Variable amounts of the 10 wt% zein stock solution (ranging from 40 g to 200 g) were then added dropwise into the respective polysaccharide solutions under identical stirring conditions (1200 rpm) to achieve final zein concentrations ranging from 1 to 5 wt%.

### 2.4. Solvent Evaporation and Post-Treatment

After preparation, all dispersions, including polysaccharide-only control samples, were kept under continuous stirring in a fume hood overnight to allow complete evaporation of ethanol. Deionized water was then added to adjust each system back to the original total mass of 400 g. All samples were stored for further characterization.

Ethanol removal was monitored gravimetrically: dispersion mass decreased from an initial 400 g to approximately 280–300 g after overnight evaporation (fume hood, continuous stirring), corresponding to a mass loss 3–4 times greater than the total ethanol introduced (~32.4 g per batch), indicating near-complete ethanol removal prior to water back-dilution to the original mass.

It is worth noting that the final bulk pH of the dispersions after ethanol evaporation and water back-dilution remained in the range of approximately 6–7 for the CMC systems and approximately 4 for the XG systems. Because the nanoparticle–polysaccharide structures are kinetically trapped upon initial anti-solvent precipitation under the originally controlled pH conditions, minor bulk pH shifts occurring afterward are not expected to substantially affect the established particle morphology.

### 2.5. Rheological Measurements

The steady-shear rheology of the fluids was measured using Fann (Fann Instrument Company, Houston, TX, USA) and Haake (ThermoFisher Scientific, Waltham, MA, USA) types of co-axial cylinder viscometers. The dimensions of the measuring geometries used, and the corresponding gap widths are given in Table 1. The Fann viscometer had 12 rotational speeds ranging from 0.9 to 600 rpm whereas the Haake viscometer had 30 rotational speeds ranging from 0.01 to 512 rpm. Both devices were calibrated using standard fluids of known viscosities prior to any measurements of test fluids. All measurements were conducted at room temperature.

For each formulation, a single dispersion was prepared, and steady-shear rheological measurements were obtained from repeated runs on that same preparation.

### 2.6. Surface Tension Measurements

The surface tension measurement of test materials (liquids) was carried out using the smartphone-based pendant-drop tensiometer supplied by Droplet Lab, Markham, ON, Canada. A pendant droplet of liquid phase is generated at the tip of a stainless-steel needle (1.8 mm diameter) connected to a 500 µL Hamilton^®^ gastight syringe (Model 1750 TPLT), Hamilton Company, Reno, NV, USA.

Each pendant droplet was allowed to stabilize for approximately 1–3 min before image capture. The high-resolution image of the pendant droplet is taken using a smartphone camera and analyzed using specialized software provided by the company. From the drop shape analysis, the software can calculate the surface tension of each solution numerically by fitting the droplet profile with the Young–Laplace equation [22]. The measurement for each test material is performed at least 15 times, and an average value is calculated. All measurements were done at room temperature.

### 2.7. SEM Procedure

The microstructural morphology of the nanocomplexes was characterized using a scanning electron microscope (SEM, Hitachi SU8700, Minato-ku, Tokyo, Japan). Prior to imaging, the freshly prepared liquid dispersions were pre-frozen at −80 °C overnight to rapidly arrest and preserve the formed network structures. Subsequently, the frozen samples were lyophilized for 24 h to completely remove water via sublimation, yielding dry powder specimens. The lyophilized powders were then carefully mounted onto aluminum stubs using double-sided conductive carbon tape. The SEM imaging was conducted at an accelerating voltage of 1.0–1.5 kV.

## 3. Results and Discussion

### 3.1. Rheology of Dispersions of Zein Nanoparticles in Sodium Carboxymethyl Cellulose

**Figure 3:**
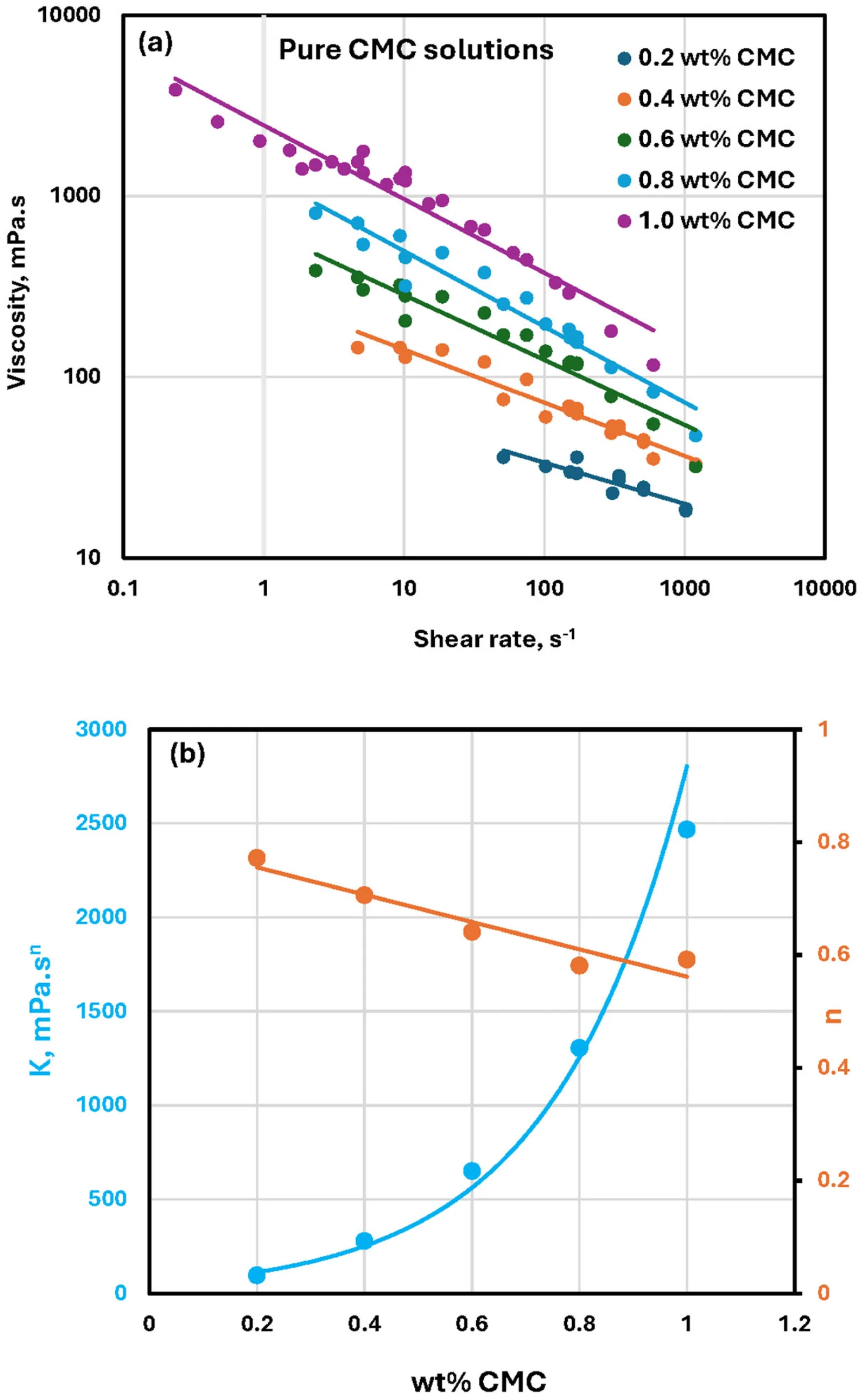
Rheological behavior of pure CMC solutions. (**a**) viscosity versus shear rate; (**b**) power-law parameters.

Figure 3 shows the steady-shear rheological data for pure CMC solutions in deionized water. The CMC solutions are shear-thinning at all CMC concentrations ranging from 0.2 to 1 wt%. The viscosity versus shear rate plot is linear on a log-log scale indicating power-law behavior:

(1) $$\tau =K{\dot{\gamma }}^{n}$$

(2) $$\eta =\frac{\tau }{\dot{\gamma }}=K{\dot{\gamma }}^{n-1}$$

where τ is shear stress, $\dot{\gamma }$ is shear rate, η is viscosity, K and n are power law parameters. The power law parameter K reflects the consistency of the material and is referred to as consistency index. The power law parameter n reflects the flow behavior of the material (Newtonian, shear thinning, shear thickening) and is referred to as flow behavior index. For Newtonian fluids, n=1 and K=η. For shear-thinning fluids, n<1. The smaller the n value, the greater the degree of shear-thinning.

The power-law parameters of CMC solutions are shown in Figure 3b. With the increase in CMC concentration, the flow behavior index n decreases linearly indicating that the CMC solution becomes more shear-thinning with the increase in CMC concentration. The consistency index K of CMC solutions increases non-linearly with the increase in CMC concentration. The increase in consistency and shear-thinning with the increase in CMC concentration is due to the entanglement and network structure formation of polymer chains.

**Figure 4:**
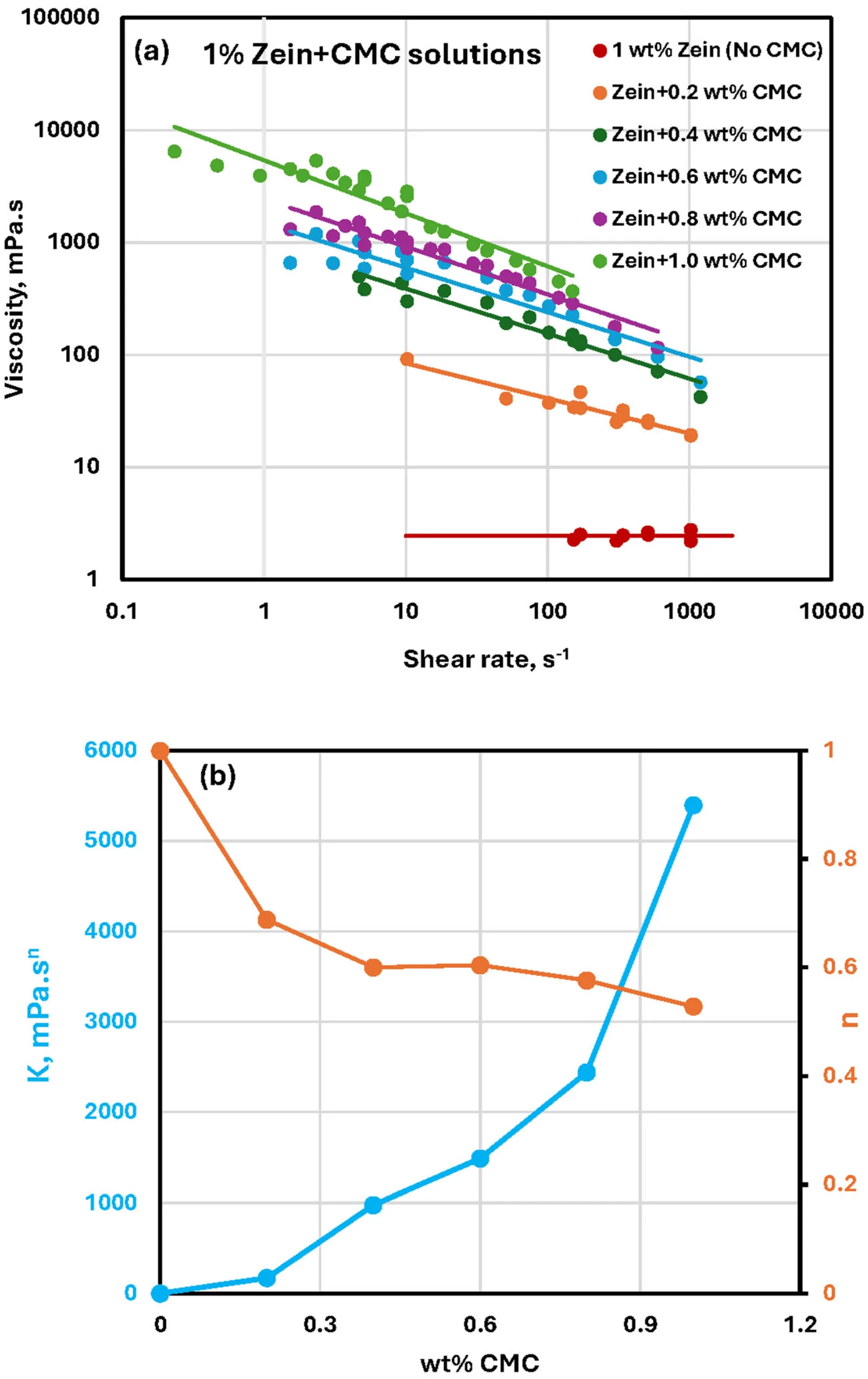
Rheological behavior of Zein–CMC dispersions. (**a**) viscosity versus shear rate; (**b**) power-law parameters.

Figure 4 shows the steady-shear rheological data for zein–CMC dispersions. The zein concentration is fixed at 1 wt% and the CMC concentration is increased from 0 to 1 wt%. While the 1 wt% zein solution is Newtonian, the zein–CMC dispersions are all shear-thinning. The power-law model describes the rheological data of zein–CMC dispersions adequately. The consistency index, K, increases substantially, and the flow behavior index, n, decreases appreciably with the increase in CMC concentration.

**Figure 5:**
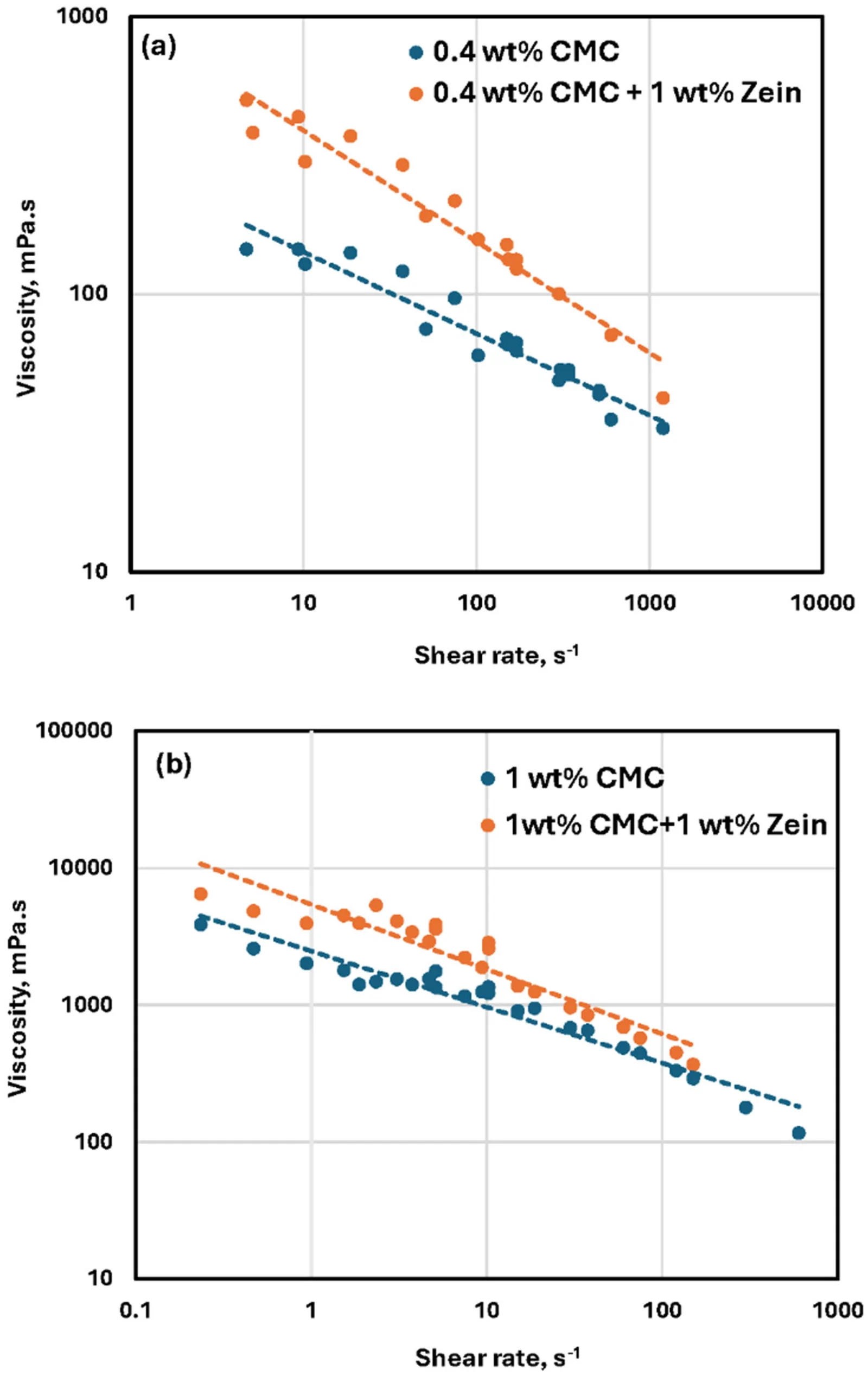
Comparison of flow curves of zein–CMC dispersions with pure CMC solutions. (**a**) 0.4 wt% CMC; (**b**) 1 wt% CMC.

**Figure 6:**
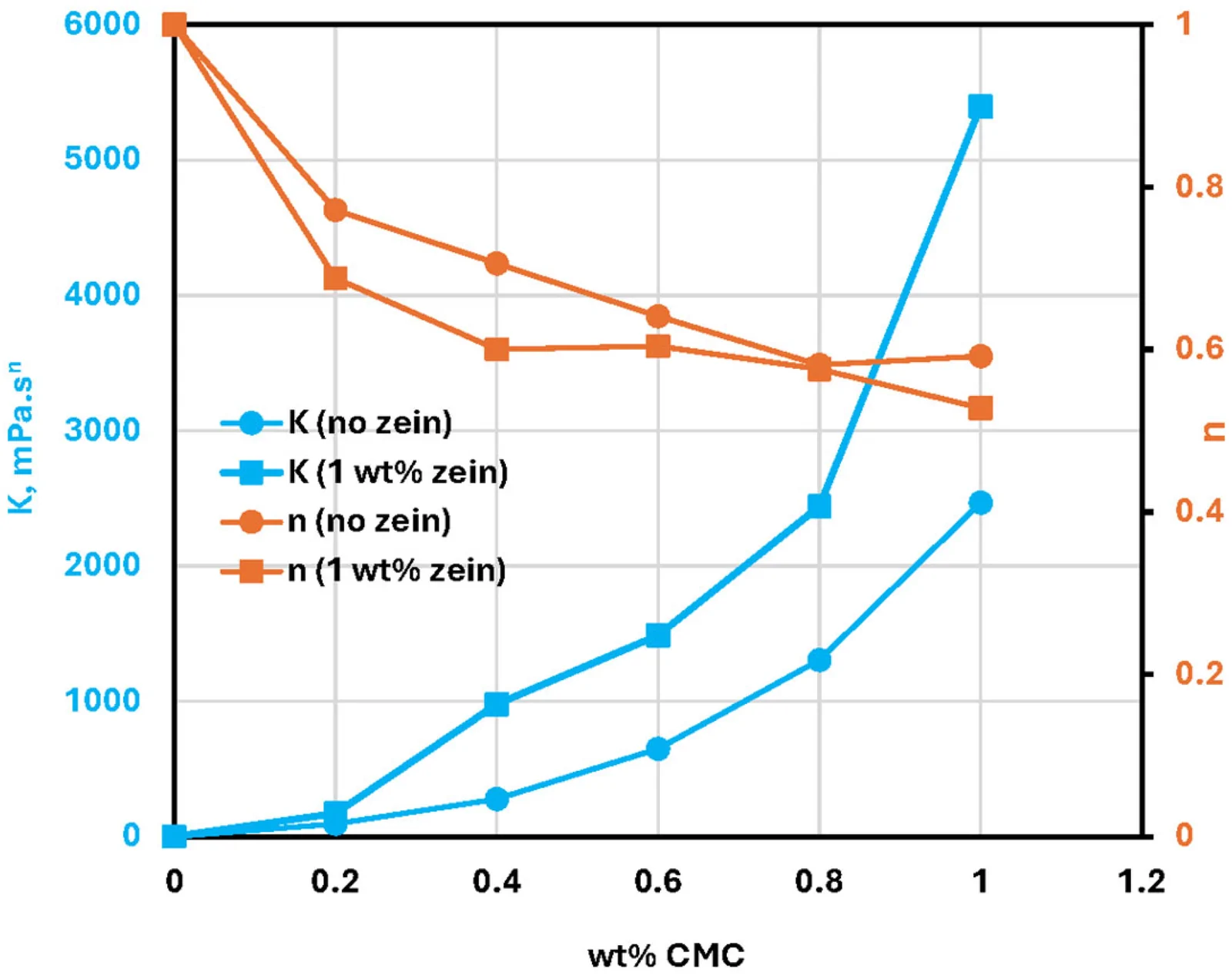
Comparison of power-law parameters (K and n) of zein–CMC dispersions with pure CMC solutions.

The viscosity of zein–CMC dispersions is significantly higher than that of pure CMC solutions, as shown in Figure 5. Figure 6 compares the consistency and flow behavior indices (K and n) of zein–CMC dispersions with those of pure CMC solutions. The consistency index of zein–CMC dispersions is higher than that of the pure CMC solutions over the entire CMC range and the gap between zein–CMC dispersion and CMC solution increases with the increase in CMC concentration. The flow behavior index of zein–CMC dispersions falls below that of the pure CMC solutions indicating greater degree of shear-thinning in zein–CMC dispersions.

**Figure 7:**
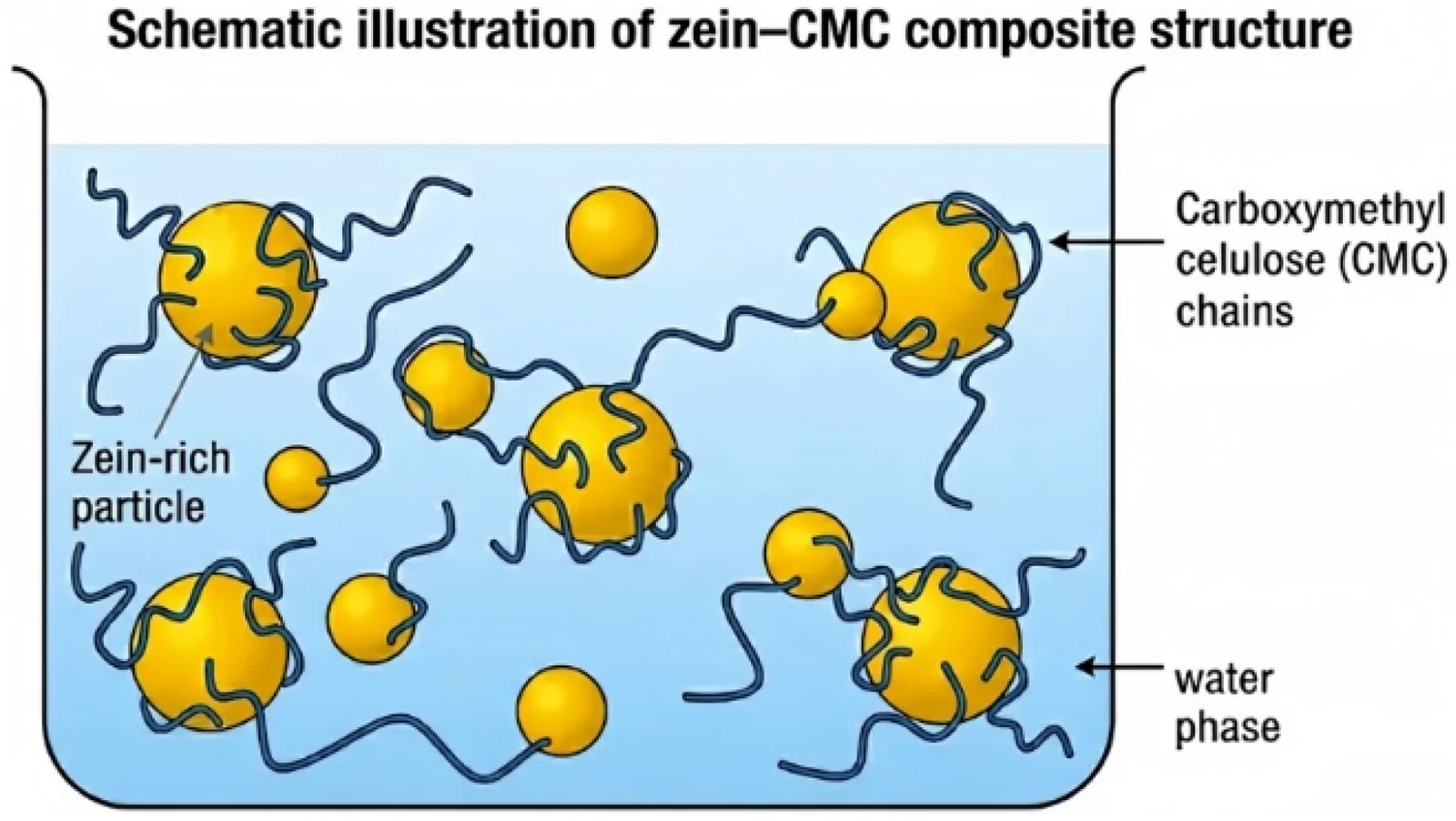
The colloidal dispersion of zein nanoparticles in CMC solution.

The change in the rheological properties (K and n) of zein–CMC dispersions in comparison with pure CMC solutions is caused by the formation and dispersion of zein nanoparticles in CMC solutions, shown schematically in Figure 7. The increase in K and the decrease in n are due to two effects: (1) the appearance of zein nanoparticles in the matrix of CMC solution. It is well known that the incorporation of solid particles in the liquid phase increases the viscosity, as demonstrated by Einstein [23] in his seminal paper, and (2) possible bridging interactions between zein nanoparticles and CMC chains, which we hypothesize contribute to the formation of a microstructure in the system (schematically illustrated in Figure 7). We note that this interpretation is inferred from the steady-shear rheological trends and has not been independently confirmed via oscillatory rheology.

**Figure 8:**
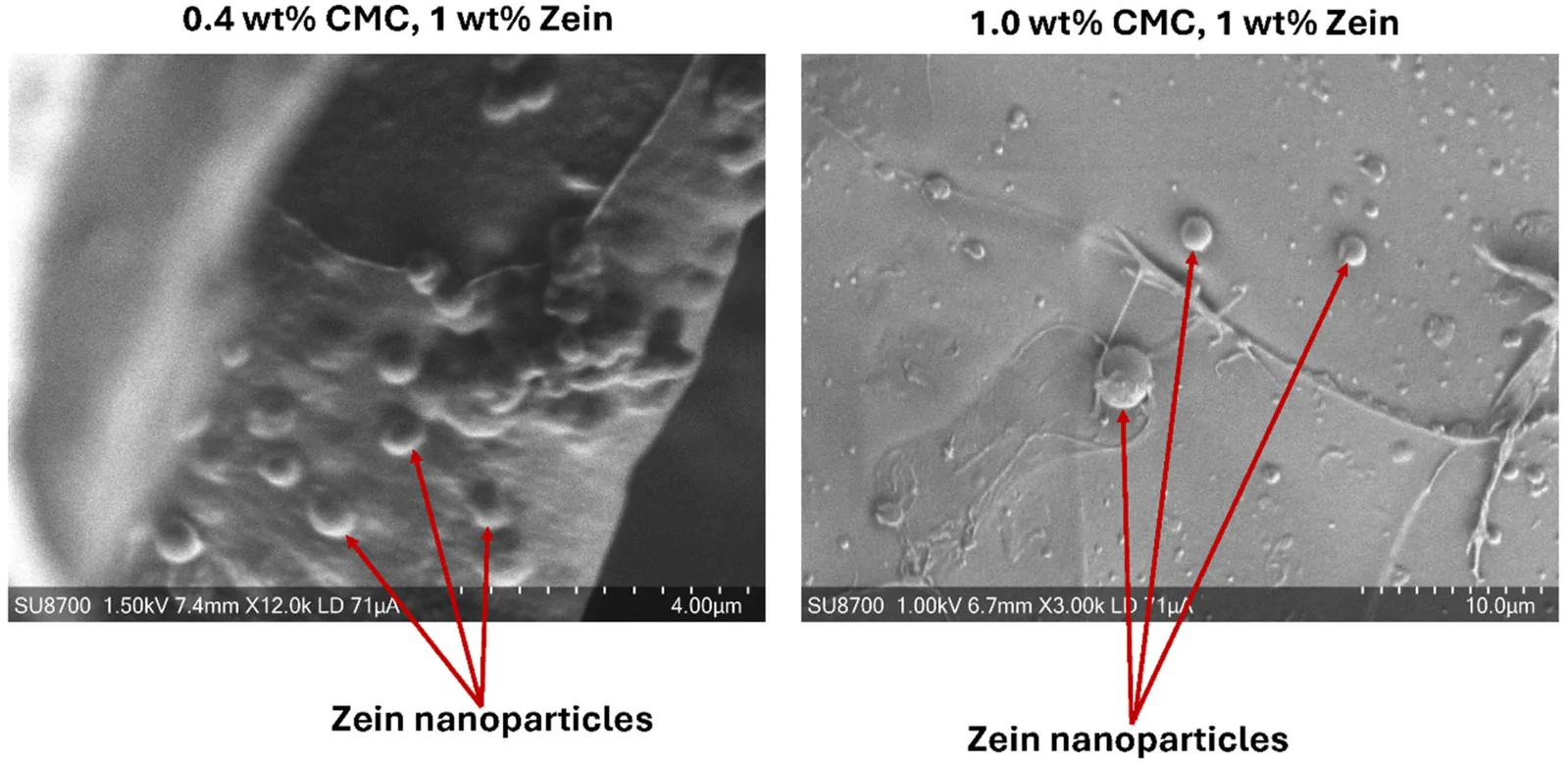
SEM images of zein–CMC dispersions at different CMC concentrations.

Figure 8 shows SEM images of zein–CMC dispersions. One can clearly see the presence of zein nanoparticles in zein–CMC systems. At a low CMC concentration of 0.4 wt%, the zein nanoparticles have diameters approximately in the range of 400 to 600 nm. At a high CMC concentration, the zein–CMC dispersion consists of many zein particles with diameters in the nm range (>150 nm) and some zein particle diameters are in the micron range (<3.5 µm). Note that, as an initial exploratory measurement of particle size, we also performed DLS (dynamic light scattering) on the 0.6 wt% CMC + zein dispersion in its native hydrated state, using five repeated measurements on the same preparation. These gave a mean Z-average particle size of approximately 2006 ± 80 nm (individual runs: 2110, 2065, 1978, 1961, and 1914 nm), which is reproducible but was flagged by the instrument software for quality considerations, likely reflecting the presence of large aggregates and/or multiple scattering effects at this sample concentration and size range; we therefore could not treat this value as a precise quantitative measurement. Qualitatively, however, the reproducible presence of micron-scale species in the hydrated dispersion is broadly consistent with our SEM observations of large particle aggregates (up to ~3.5 µm) at correspondingly high CMC concentrations, suggesting that the aggregate structures observed by SEM are not artifacts of the freeze-drying process.

It should also be mentioned that we carried out the ζ-potential measurement of the 0.6 wt% CMC + zein dispersion (unadjusted, near-neutral pH). Five repeated measurements on the same preparation gave a mean value of −55.8 ± 1.5 mV (individual runs: −56.2, −53.8, −54.7, −57.1, and −57.3 mV; Zetasizer quality flag: Good for all runs), indicating a highly reproducible, strongly negative surface charge. This is consistent with a CMC-dominated surface charge, as would be expected if CMC chains associate with and coat the zein nanoparticle surface. This also supports the bridging of zein nanoparticles with CMC chains.

#### Effect of Zein Concentration on the Rheology of Dispersions of Zein Nanoparticles in Sodium Carboxymethyl Cellulose

**Figure 9:**
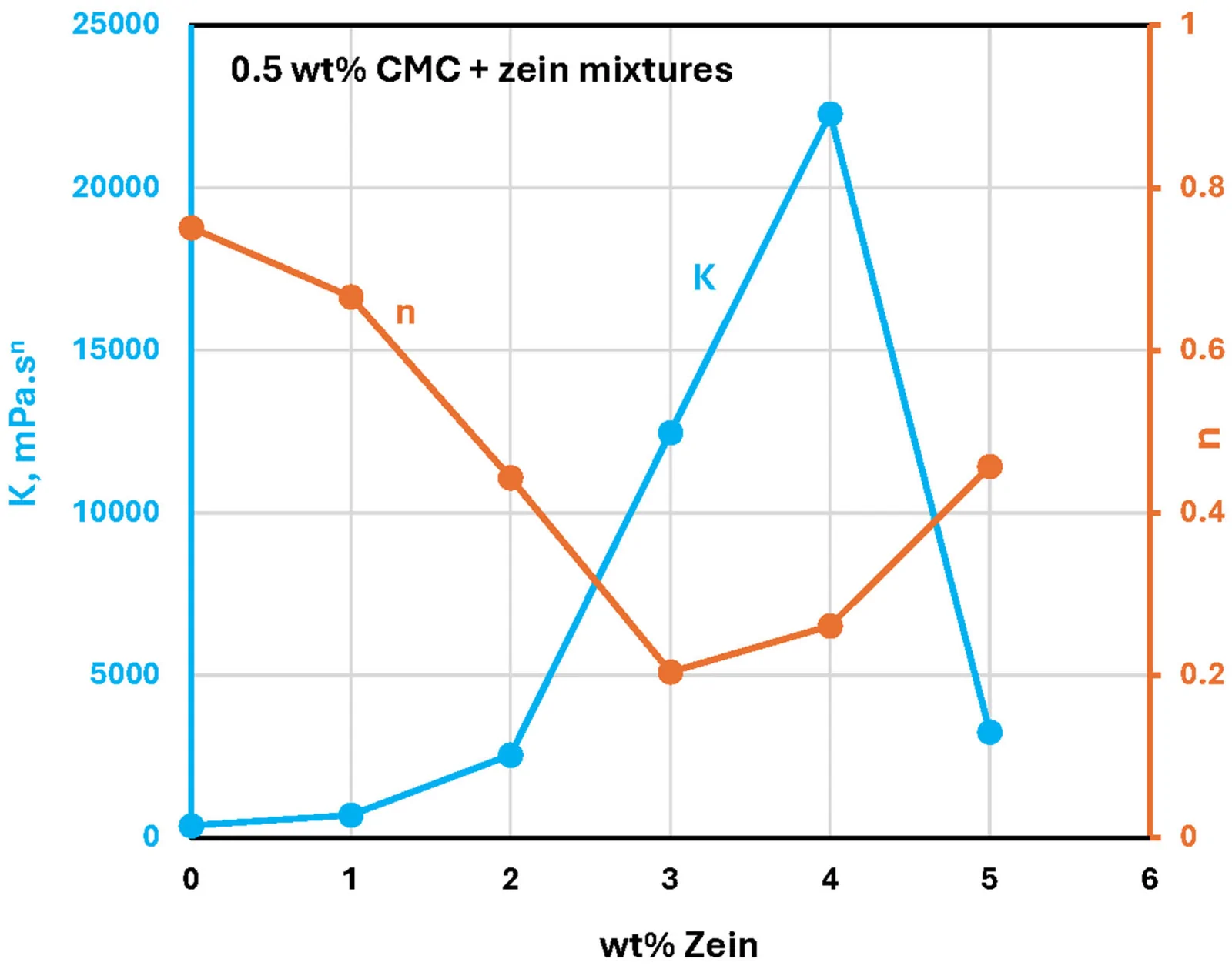
Effect of zein concentration on the rheological properties of zein–CMC dispersions at a fixed CMC concentration of 0.5 wt%.

The zein concentration of the dispersion of zein nanoparticles in carboxymethyl cellulose varied from 0 to 5 wt% at a fixed CMC concentration of 0.5 wt%. Figure 9 shows the effect of zein concentration on the rheological properties of dispersion of zein nanoparticles in CMC. With the increase in zein concentration from 0 to 4 wt%, the consistency increased substantially especially in the zein concentration range of 2 to 4 wt%. The flow behavior index, n, decreased simultaneously, indicating an increase in the degree of shear-thinning. This is not surprising, as the concentration of zein nanoparticles of the dispersion increases with the increase in zein concentration. The increase in the nanoparticle volume fraction is expected to increase the viscosity of dispersion in accordance with the Einstein law for the viscosity of suspensions. Furthermore, the increase in the zein nanoparticle concentration reinforces bridging of nanoparticles and the microstructure of zein nanoparticles and CMC polymer chains.

**Figure 10:**
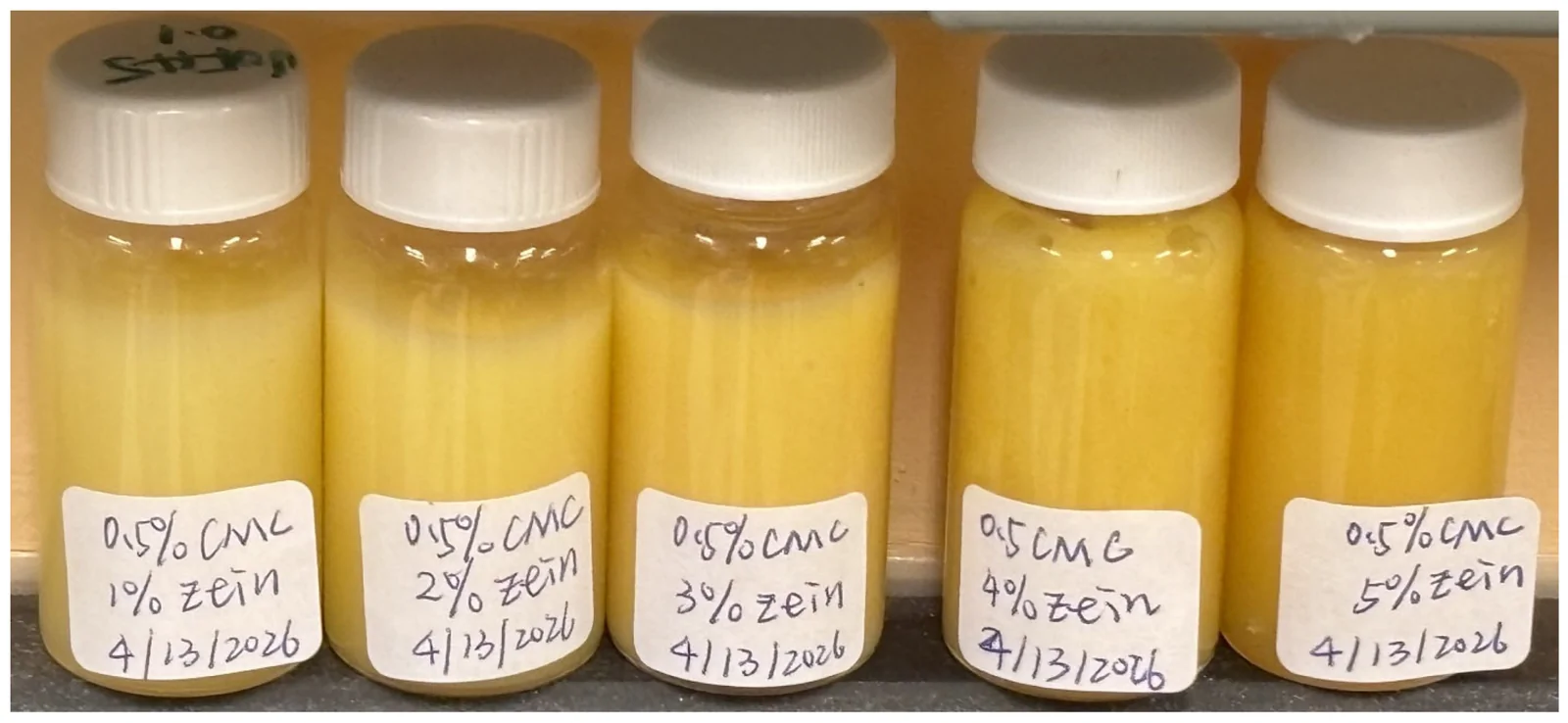
Appearance of zein–CMC dispersions at different zein concentrations with CMC concentration fixed at 0.5 wt%.

Interestingly, a sharp drop in consistency occurs as the zein concentration is increased from 4 to 5 wt%. This is consistent with a disruption of the zein nanoparticle–CMC microstructure at high zein concentration, as is inferred from the sharp drop in consistency index; we note that this interpretation is based on steady-shear behavior alone. This may be due to aggregation of zein nanoparticles at high zein concentration, although the exact reason is not known at present. It should, however, be noted that the dispersion of zein nanoparticles was still homogeneous with no evidence of any phase separation (see Figure 10).

### 3.2. Surface Tension of Dispersions of Zein Nanoparticles in Sodium Carboxymethyl Cellulose

**Figure 11:**
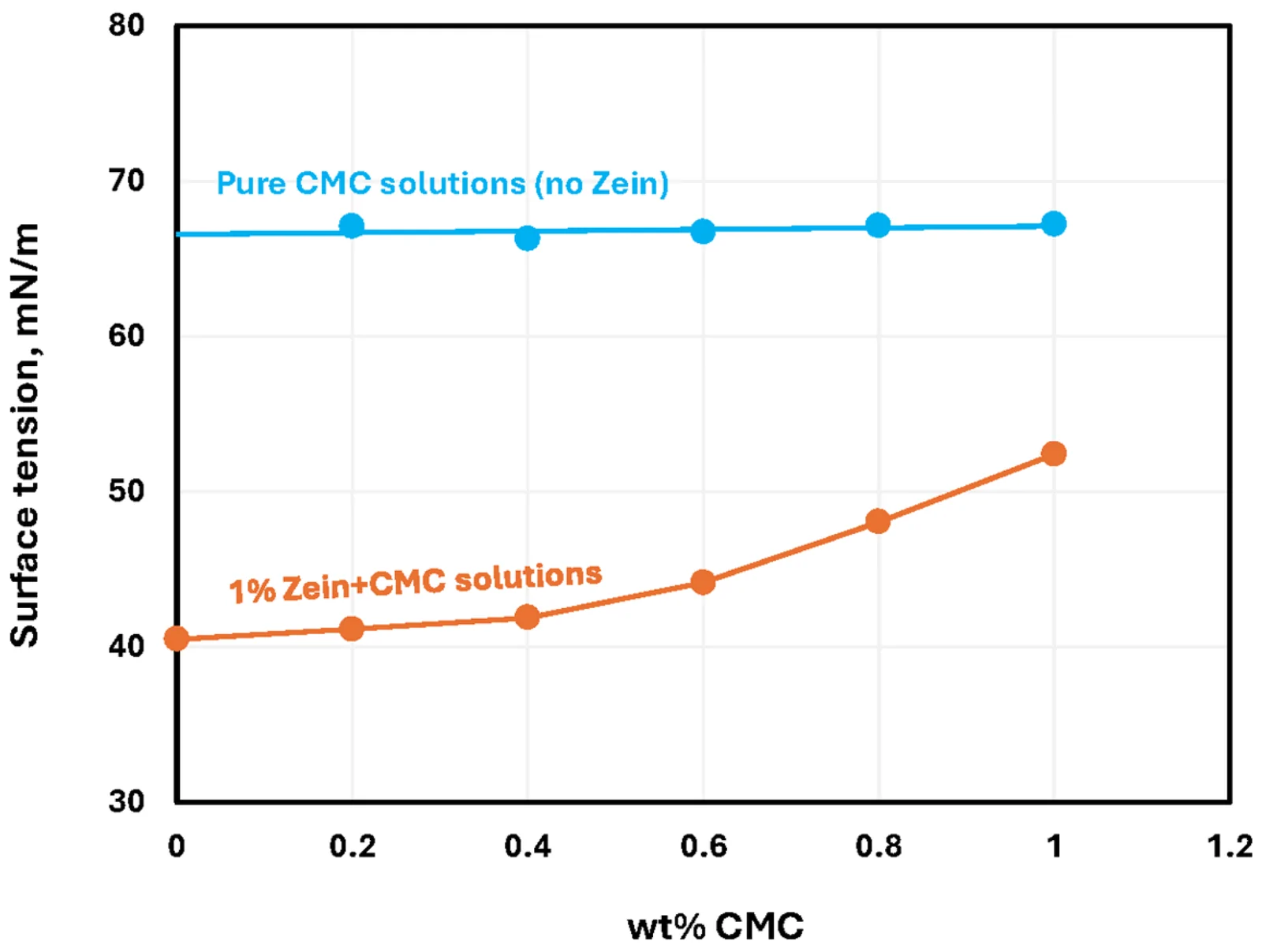
Surface tension of pure CMC solutions (no zein) and zein–CMC dispersions as function of CMC concentration.

**Figure 12:**
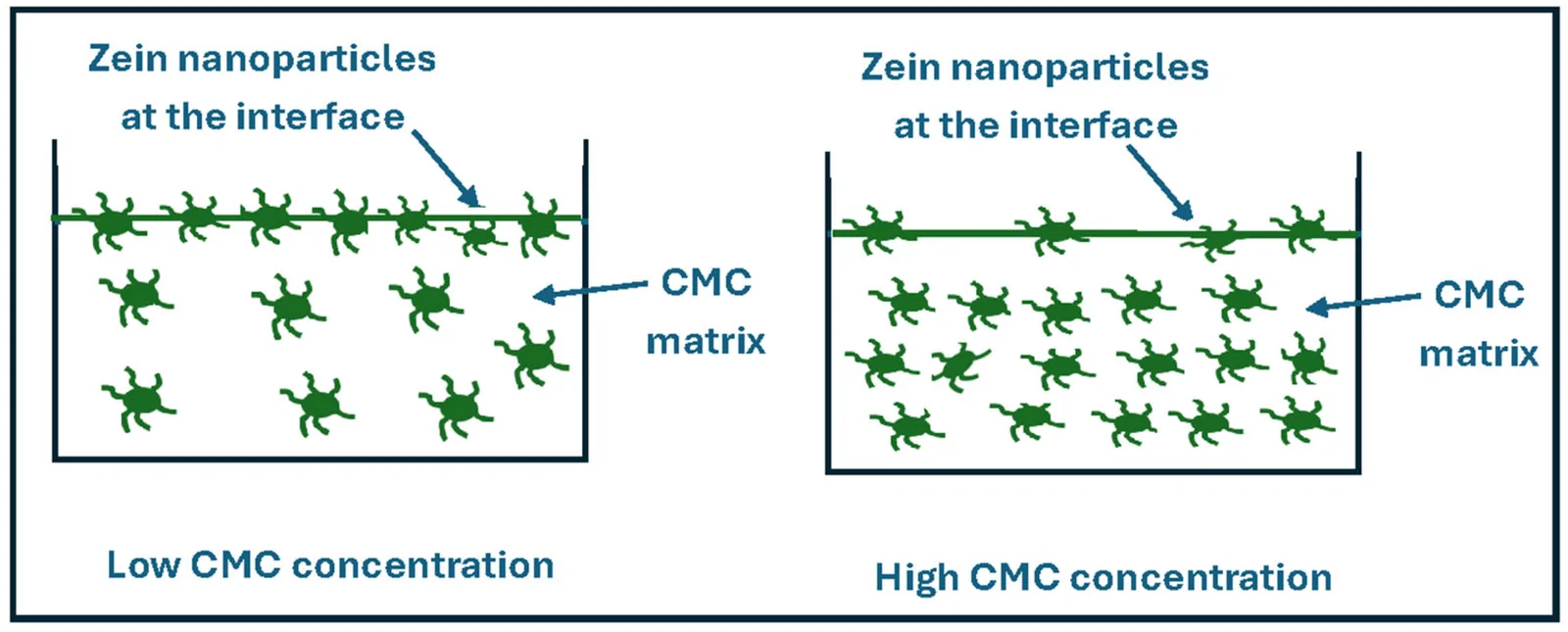
Adsorption of zein nanoparticles at the air/liquid interface at low and high concentrations of CMC.

Figure 11 shows the variations of surface tension with the CMC concentration of pure CMC solutions and dispersions of zein nanoparticles in CMC at a fixed zein concentration of 1 wt%. The pure CMC solutions have a constant surface tension independent of CMC concentration indicating that pure CMC is not surface-active. With the addition 1 wt% of zein nanoparticles in the zein–CMC dispersions, the surface tension drops substantially indicating that as the zein nanoparticles are surface-active. Clearly the surface-active zein-derived species (potentially including intact nanoparticles, dissociated protein, or protein–polysaccharide complexes) adsorb at the air/liquid interface, causing a sharp drop in the surface tension. With the increase in CMC concentration at a fixed zein nanoparticle concentration of 1 wt%, the surface tension increases probably because the nanoparticles remain trapped in the bulk liquid and a smaller number of zein nanoparticles get adsorbed at the air/liquid interface. This is shown schematically in Figure 12. It should be noted that the surface tension values shown in Figure 11 (and in subsequent surface tension figures) represent the average of 15 repeated pendant-drop measurements taken from a single prepared solution.

**Figure 13:**
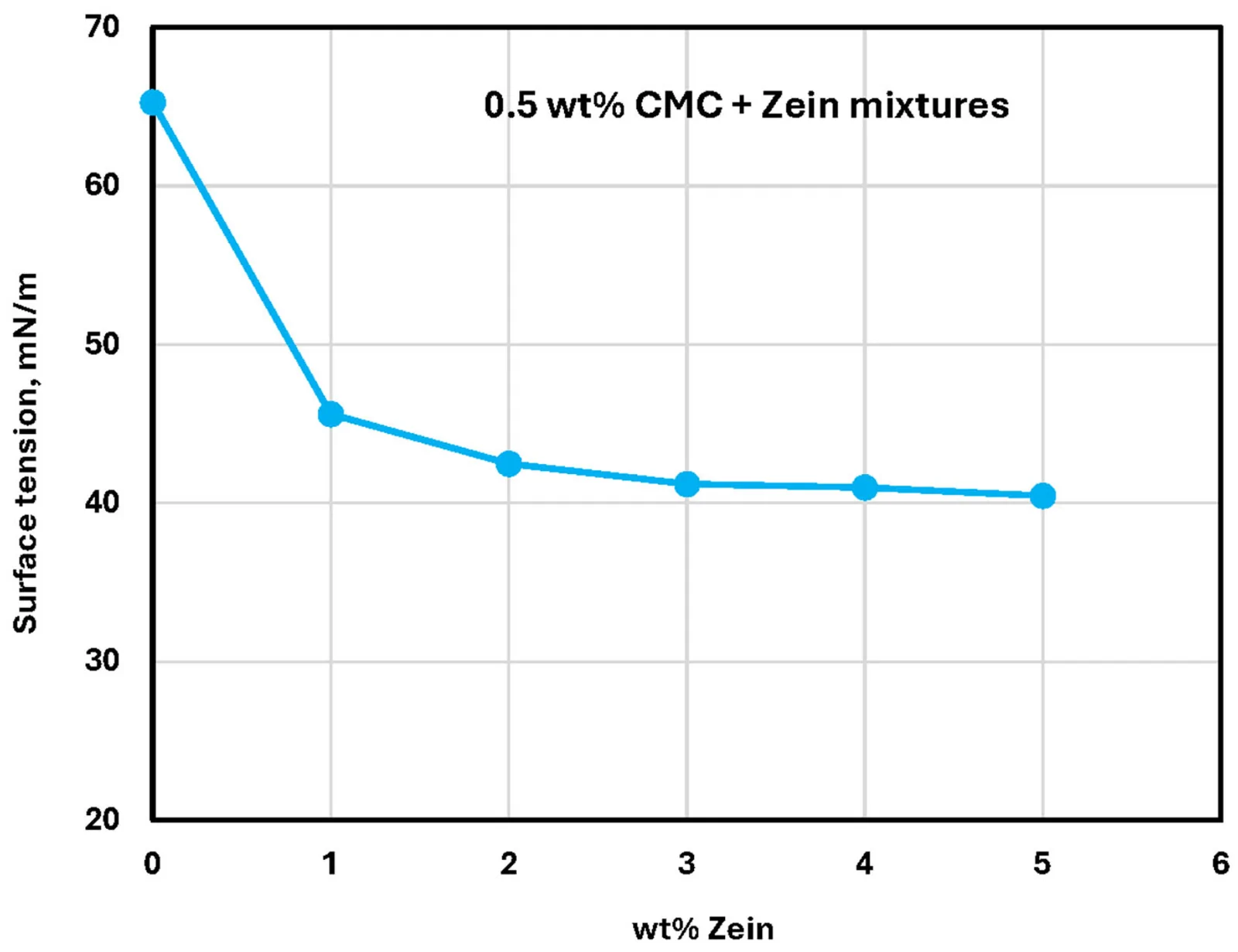
Effect of zein concentration on the surface tension of zein–CMC dispersions at a fixed CMC concentration of 0.5 wt%.

Figure 13 shows the effect of zein concentration on the surface tension of zein–CMC dispersions at a fixed CMC concentration of 0.5 wt%. The surface tension drops sharply with the increase in the zein concentration from 0 to 1 wt%. With a further increase in the zein concentration, the surface tension decreases slowly and levels off beyond 3 wt% zein. The decrease in surface tension is due to the increase in adsorption of zein nanoparticles at the air/liquid interface. At high zein concentrations, the interface becomes saturated with zein nanoparticles and hence, the surface tension levels off.

### 3.3. Rheology of Dispersions of Zein Nanoparticles in Xanthan Gum

**Figure 14:**
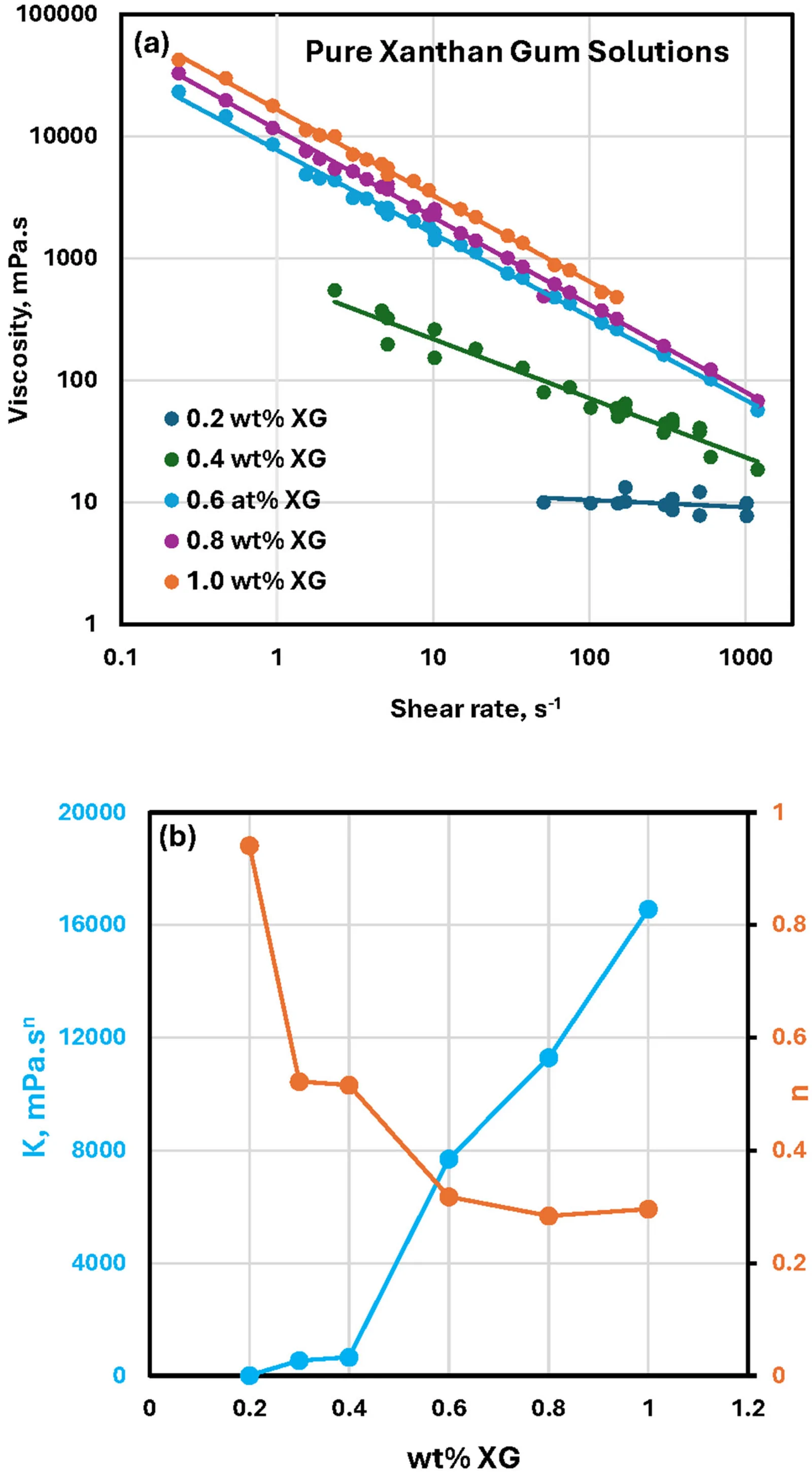
Rheological behavior of pure XG solutions. (**a**) viscosity versus shear rate; (**b**) power-law parameters.

**Figure 15:**
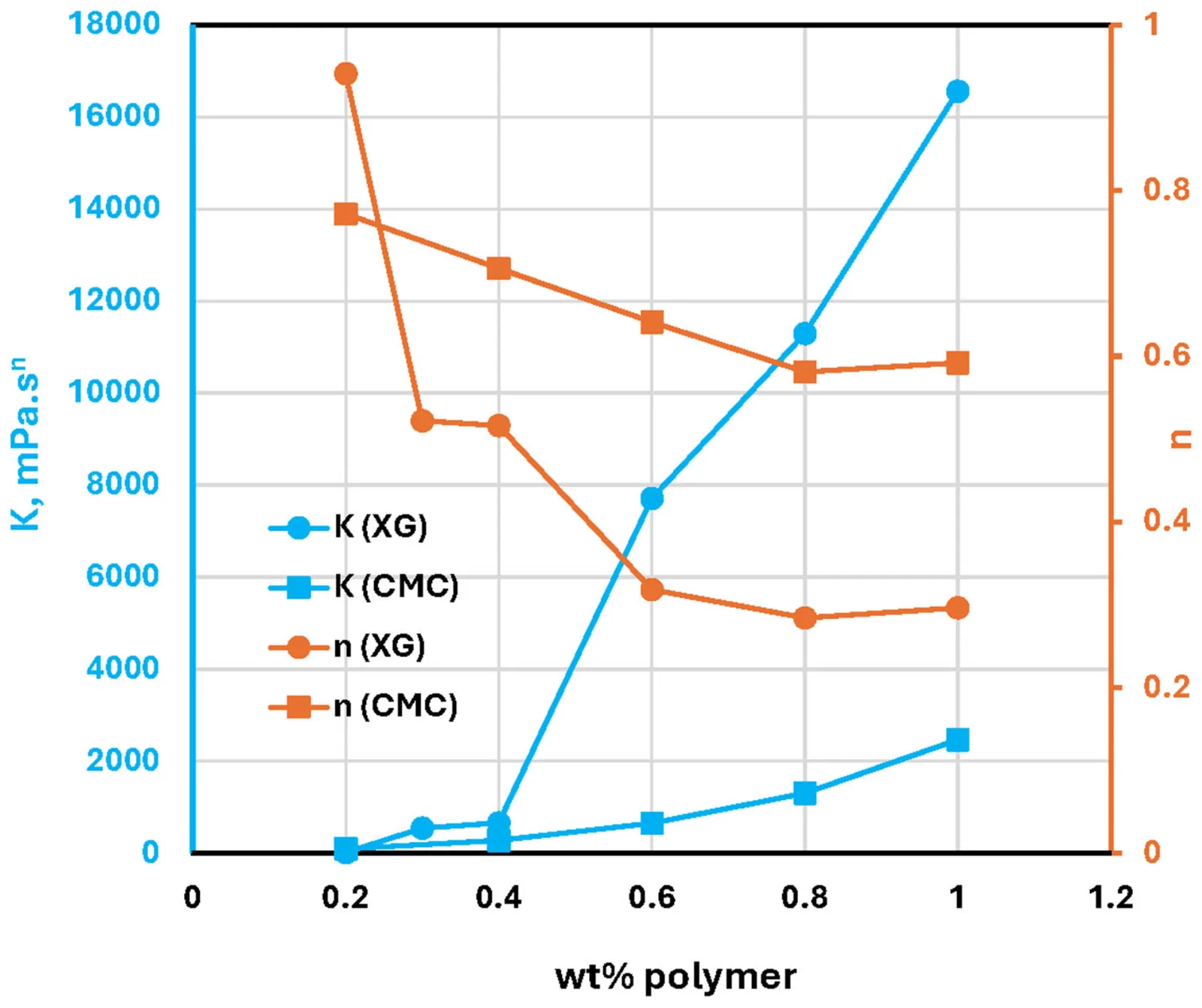
Comparison of power-law parameters of pure CMC and XG solutions.

Figure 14 shows the steady-shear rheological data for pure xanthan gum solutions in deionized water, without any zein. Like CMC solutions, XG solutions are also shear-thinning at all XG concentrations ranging from 0.2 to 1 wt%. The plots of viscosity versus shear rate are linear on a log-log scale indicating power-law behavior like CMC solutions. The consistency index K rises sharply above XG concentration of 0.4 wt%. The flow behavior index n decreases sharply when XG concentration is increased from 0.2 to 0.6 wt% and levels off at higher XG concentration. It is important to recognize that XG solutions are much more viscous and shear-thinning than CMC solutions. The comparison of the power-law parameters of pure CMC and XG solutions is shown in Figure 15. The consistency index K of XG solution is high almost 8 times that of CMC solution at a polymer concentration of 1 wt%. The flow behavior index n of XG solution is almost one-half of CMC solution at a polymer concentration of 1 wt%. Note that lower the flow behavior index greater is the degree of shear-thinning. Xanthan gum solutions are more viscous and shear-thinning, likely due to the presence of anionic side chains on XG molecules. These side chains of the neighboring xanthan molecules interfere with each other resulting in decreased mobility of xanthan molecules.

It is worth noting that the literature values of the polymer overlap concentration (c*) for CMC and XG of comparable molecular weight (approximately 0.1 wt% and 0.005–0.008 wt%, respectively [24]) fall well below the lowest polysaccharide concentration examined in this study (0.2 wt%), indicating that the polysaccharide chains in both systems are already in the semi-dilute/entangled regime across the concentration range studied. Zein nanoparticles are therefore introduced into an already-entangled polysaccharide matrix, and the concentration-dependent rheological changes reflect how this pre-existing network responds to the presence of zein.

**Figure 16:**
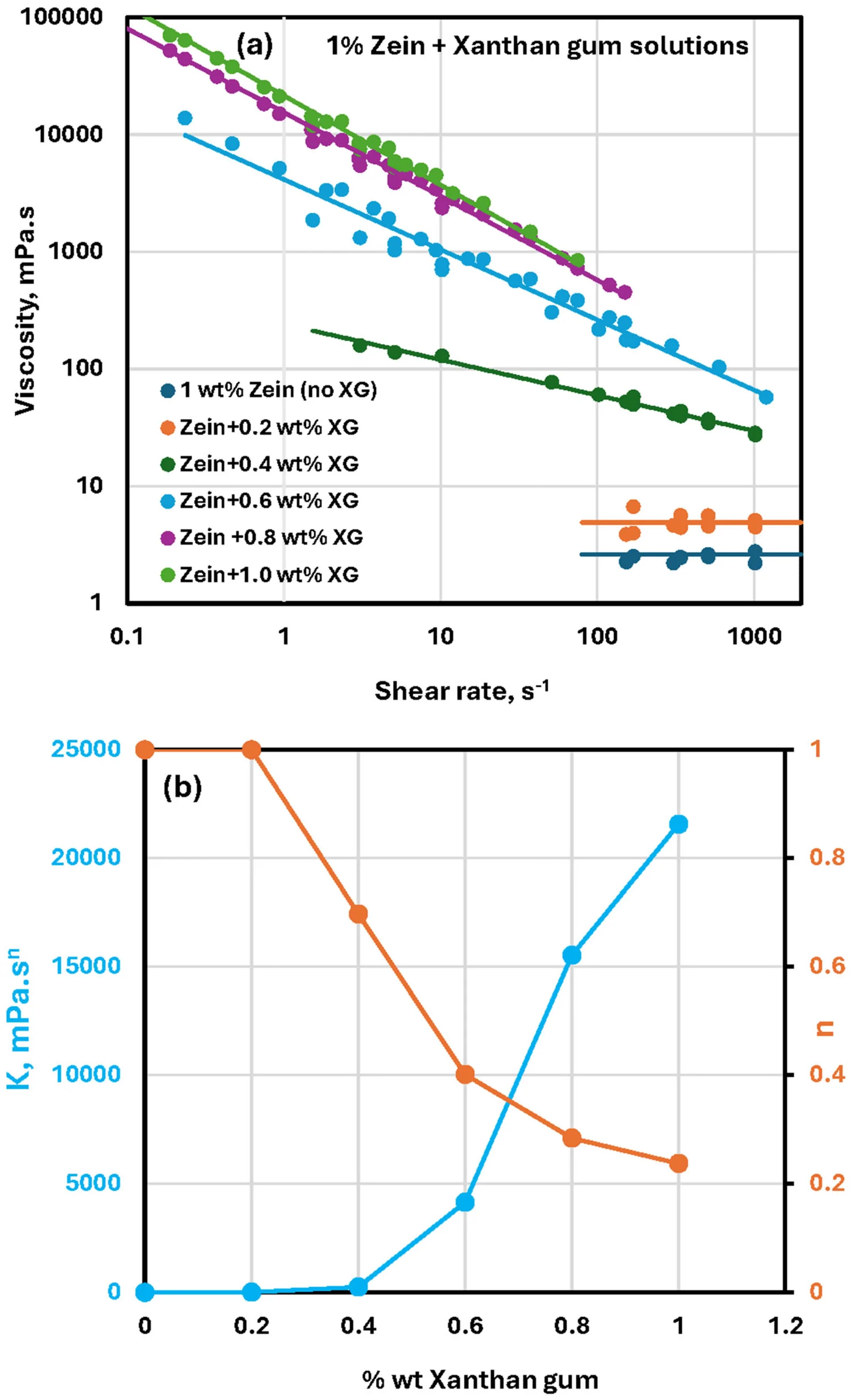
Rheological behavior of zein–XG dispersions. (**a**) viscosity versus shear rate; (**b**) power-law parameters.

The rheological behavior of zein–XG dispersions is shown in Figure 16. The zein concentration is fixed at 1 wt%, and the XG concentration is increased from 0 to 1 wt%. While the 1 wt% zein solution without any XG and the zein–XG dispersion at 0.2 wt% XG are Newtonian, the zein–XG dispersions at XG concentrations above 0.2 wt% are all shear-thinning. The power-law model describes the rheological data of zein–XG dispersions adequately. The consistency index, K, increases substantially, and the flow behavior index, n, decreases appreciably with the increase in the XG concentration above 0.4 wt%. A similar behavior was observed in the case of zein–CMC dispersions (see Figure 4).

**Figure 17:**
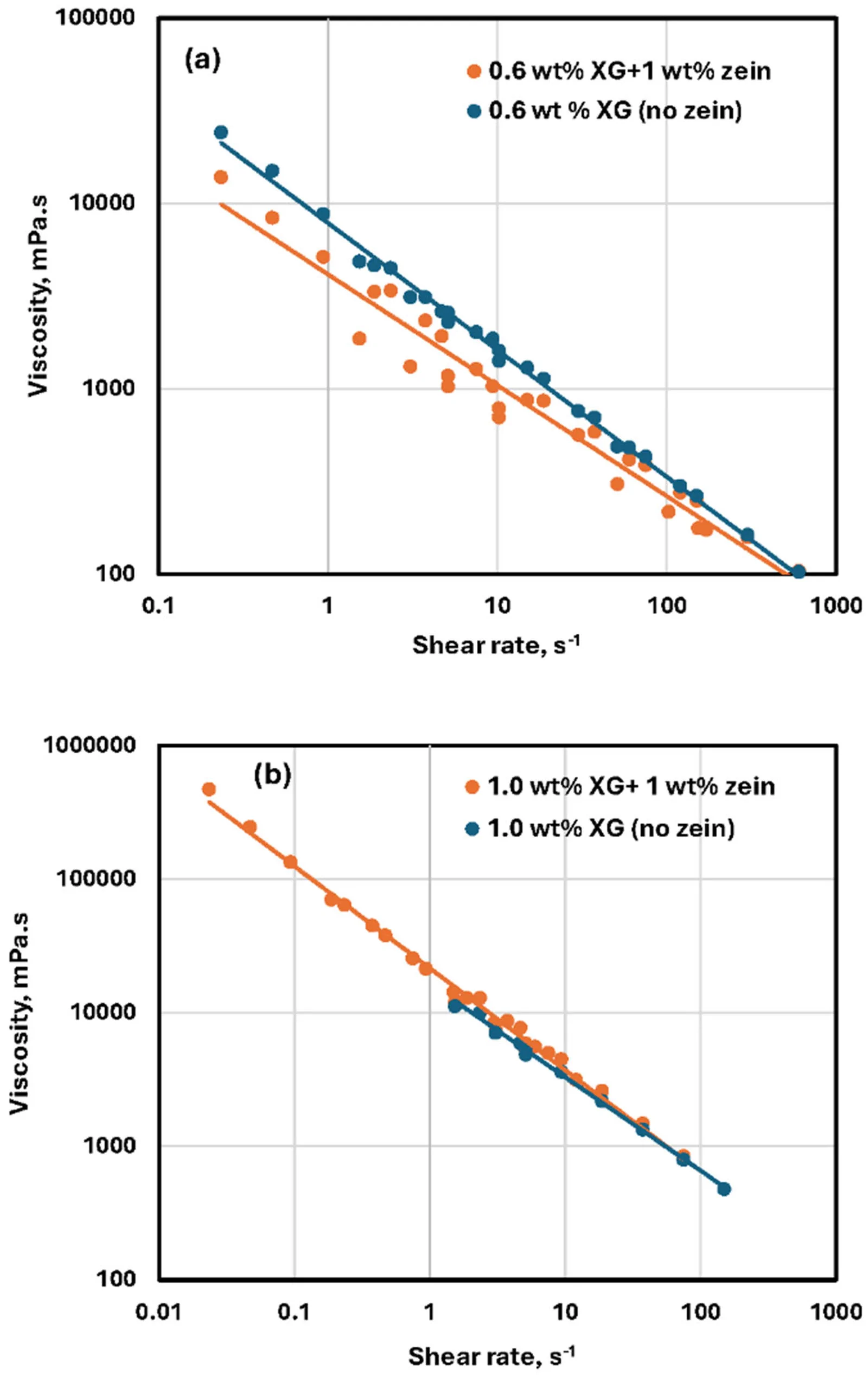
Comparison of flow curves of zein–XG dispersions with pure XG solutions. (**a**) 0.6 wt% XG; (**b**) 1 wt% XG.

**Figure 18:**
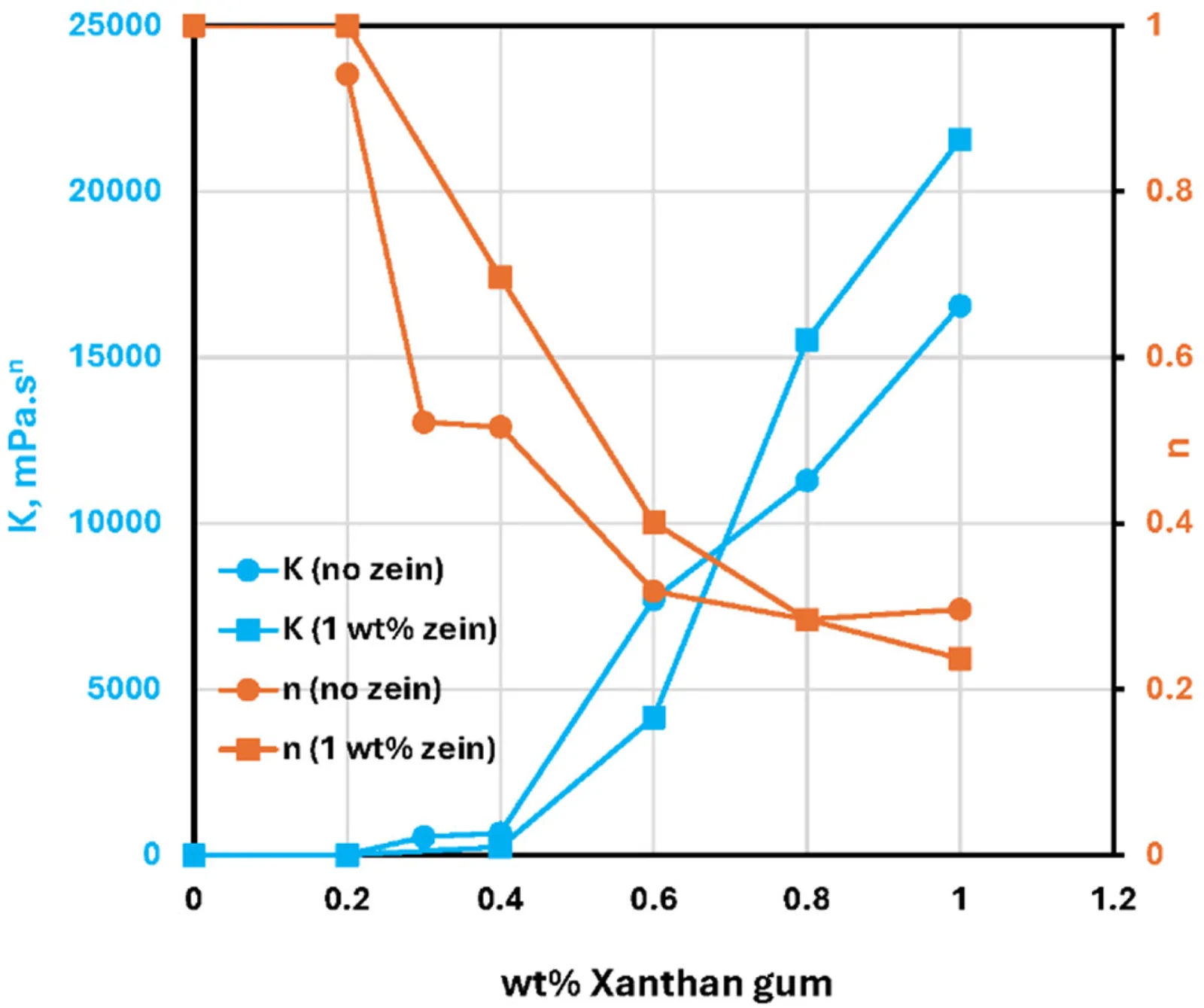
Comparison of power-law parameters (K and n) of zein-XG dispersions with pure XG solutions.

Figure 17 compares the viscosity of zein–XG dispersions with pure XG solutions. Unlike zein–CMC dispersions, the viscosity of zein–XG dispersions decreases with the incorporation of zein nanoparticles at a XG concentration of 0.6 wt%. However, at a high XG concentration of 1 wt%, the opposite behavior is observed in that the viscosity of zein–XG dispersions increases with the addition of zein nanoparticles. Figure 18 compares the consistency and flow behavior indices (K and n) of zein-XG dispersions with those of pure XG solutions. The consistency index of zein–XG dispersions is lower than that of the pure XG solutions over the XG range of approximately 0 to 0.7 wt%. At higher XG concentrations (>0.7 wt%), the consistency index of zein–XG dispersions is higher than that of the pure XG solutions. At XG concentrations of less than about 0.8 wt%, the zein-XG dispersions tend to become less shear-thinning (n increases). However, at a higher XG concentration of 1 wt%, the zein-XG dispersion becomes more shear-thinning (n decreases).

**Figure 19:**
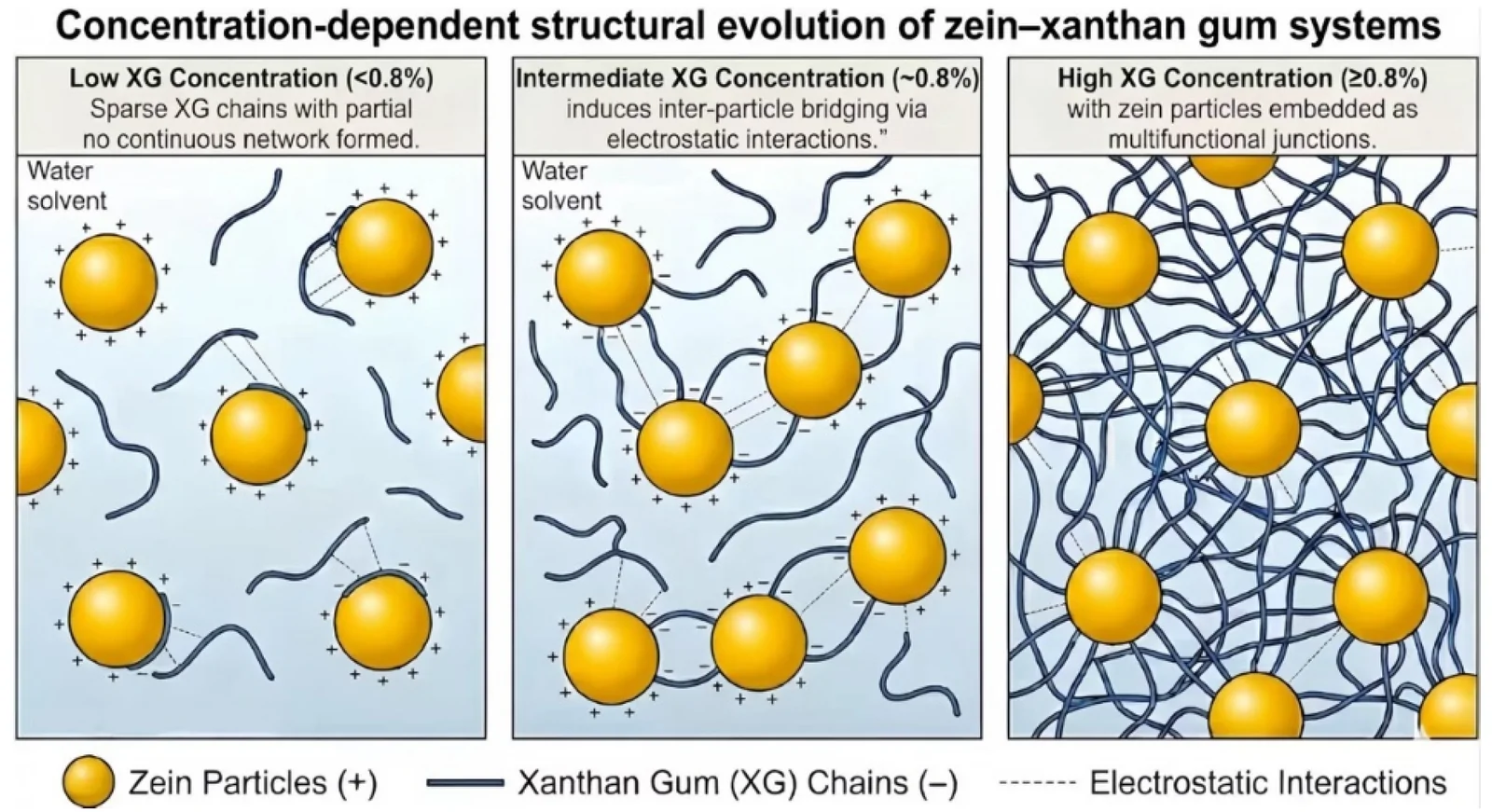
Microstructural transition of zein–XG composite networks.

It appears that the internal morphology of zein–XG dispersions exhibits a distinct concentration-dependent structural evolution, as schematically illustrated in Figure 19. At low XG concentrations (<0.8 wt%), zein–XG complexation leads to adsorption of XG onto zein nanoparticles, consistent with a reduction in the effective polymer concentration available for chain entanglement. At higher concentrations (≥0.8 wt%), we hypothesize that zein nanoparticles may act as junction points bridging XG chains, which would be consistent with the sharp increase in viscosity observed. This concentration-dependent transition is consistent with previous reports showing a pronounced increase in viscosity at ~0.8 wt% XG in zein–XG systems [25,26].

**Figure 20:**
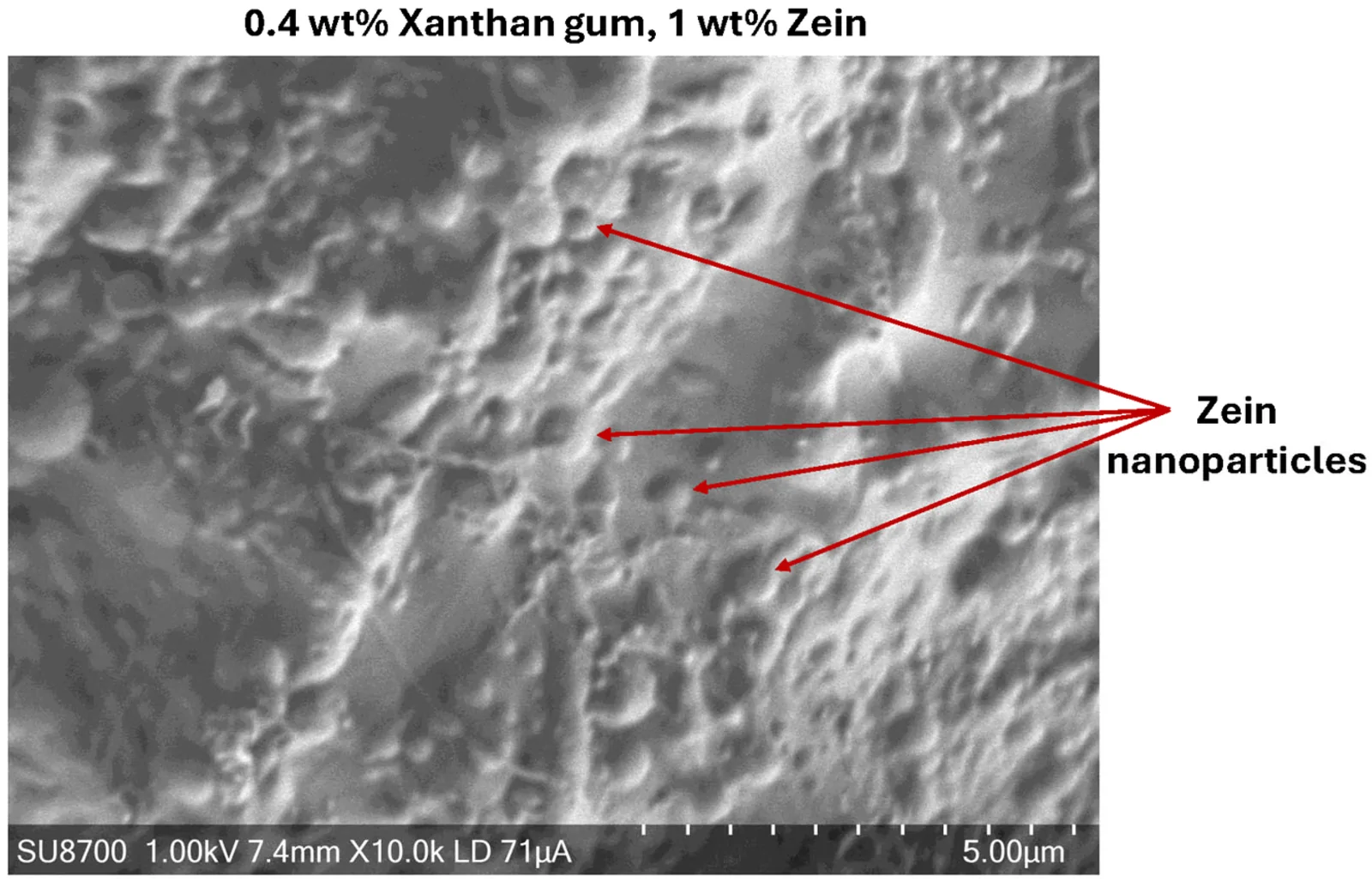
SEM image of zein–XG dispersion at XG concentration of 0.4 wt%.

Figure 20 shows an SEM image of zein–XG dispersion at 0.4 wt% XG. Zein nanoparticles can be seen clearly. The zein nanoparticles have diameters approximately in the range of 175 to 600 nm. The zein nanoparticle size of zein–XG dispersion is like that of zein–CMC dispersion.

#### Effect of Zein Concentration on the Rheology of Dispersions of Zein Nanoparticles in Xanthan Gum

**Figure 21:**
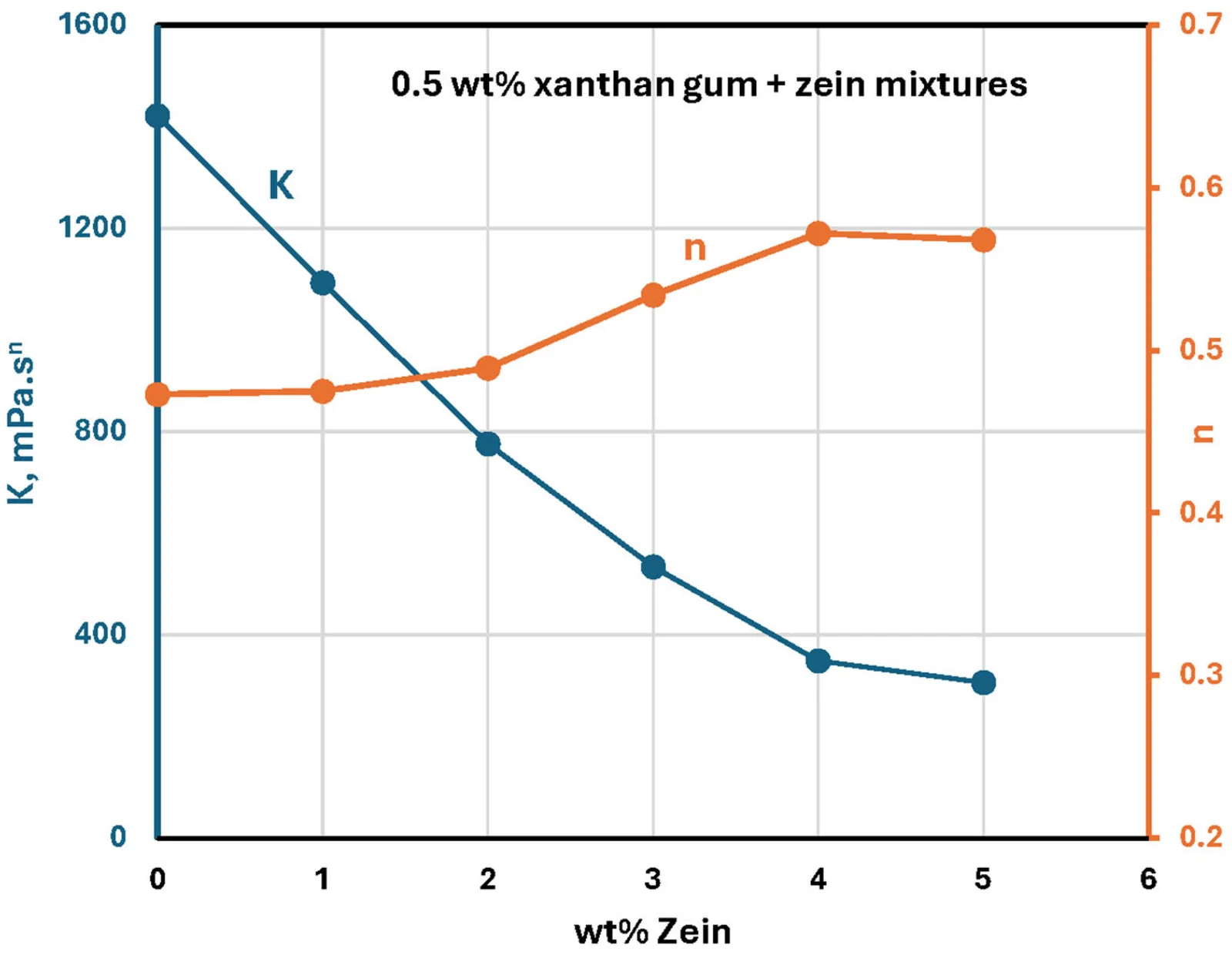
Effect of zein concentration on the rheological properties of zein–XG dispersions at a fixed XG concentration of 0.5 wt%.

**Figure 22:**
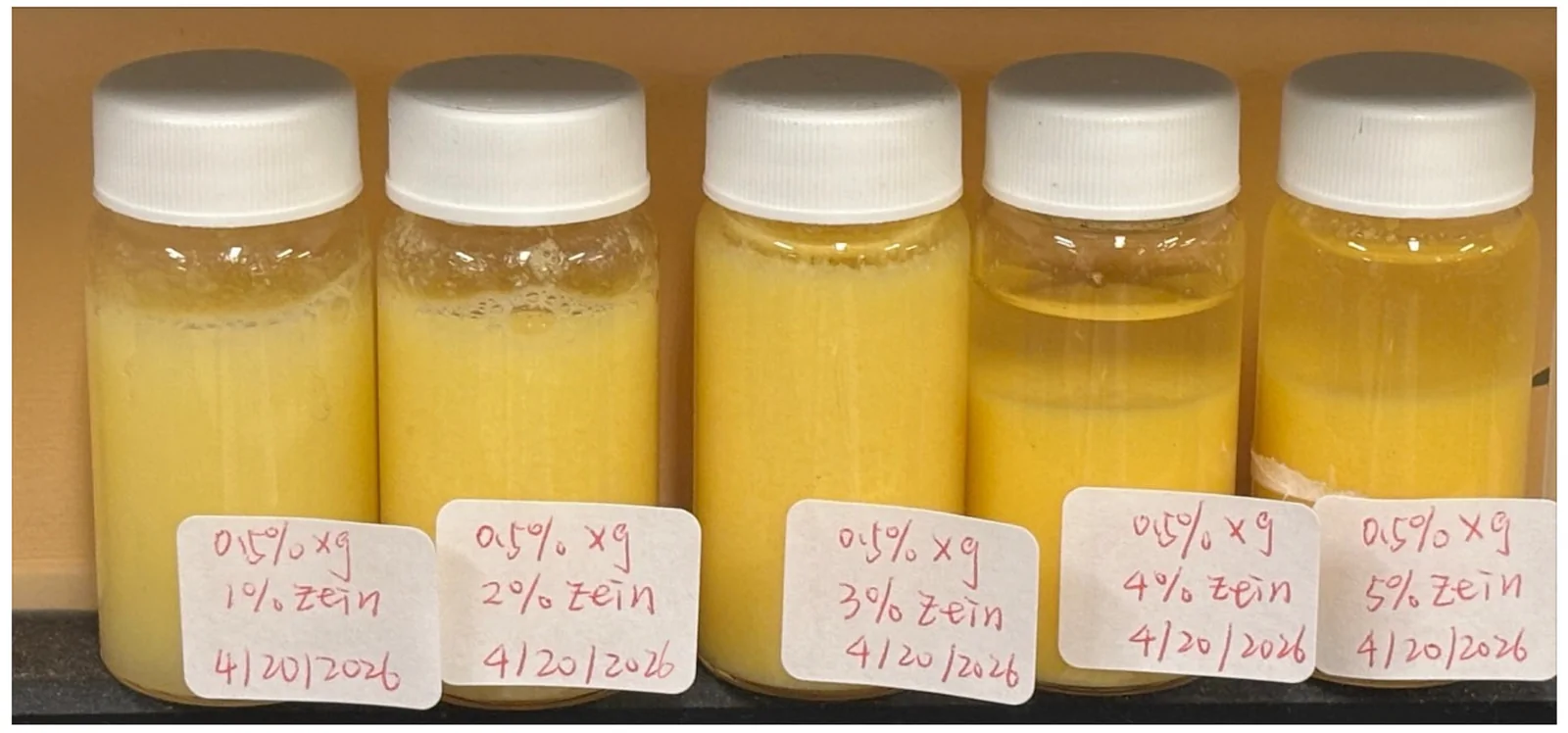
Appearance of zein–XG dispersions at different zein concentrations with XG concentration fixed at 0.5 wt%.

Figure 21 shows the effect of zein concentration on the rheological properties of dispersion of zein nanoparticles in xanthan gum. The XG concentration is fixed at 0.5 wt%, and the zein concentration of the dispersion varied from 0 to 5 wt%. With the increase in the zein concentration, the consistency decreases over the full zein concentration range from 0 to 5 wt%. The flow behavior index n rises simultaneously, indicating a decrease in the degree of shear-thinning. This behavior is opposite to what was observed in the case of zein–CMC dispersions. Furthermore, the zein–XG dispersion undergoes phase separation at zein concentrations larger than 4 wt%, as shown in the pictures of Figure 22.

**Figure 23:**
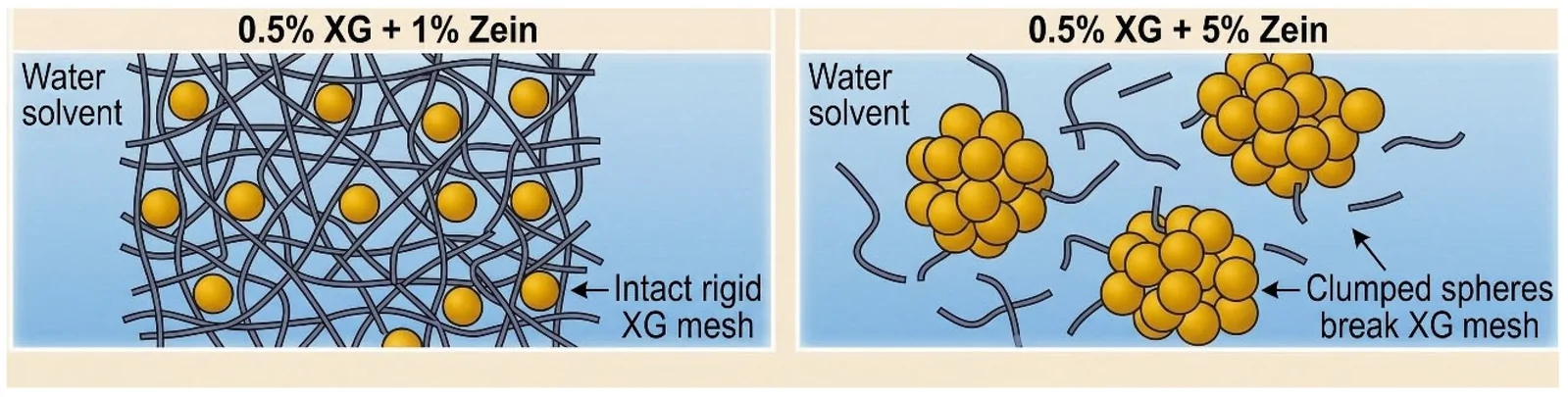
Clumping/clustering of zein nanoparticles with the increase in zein concentration.

**Figure 24:**
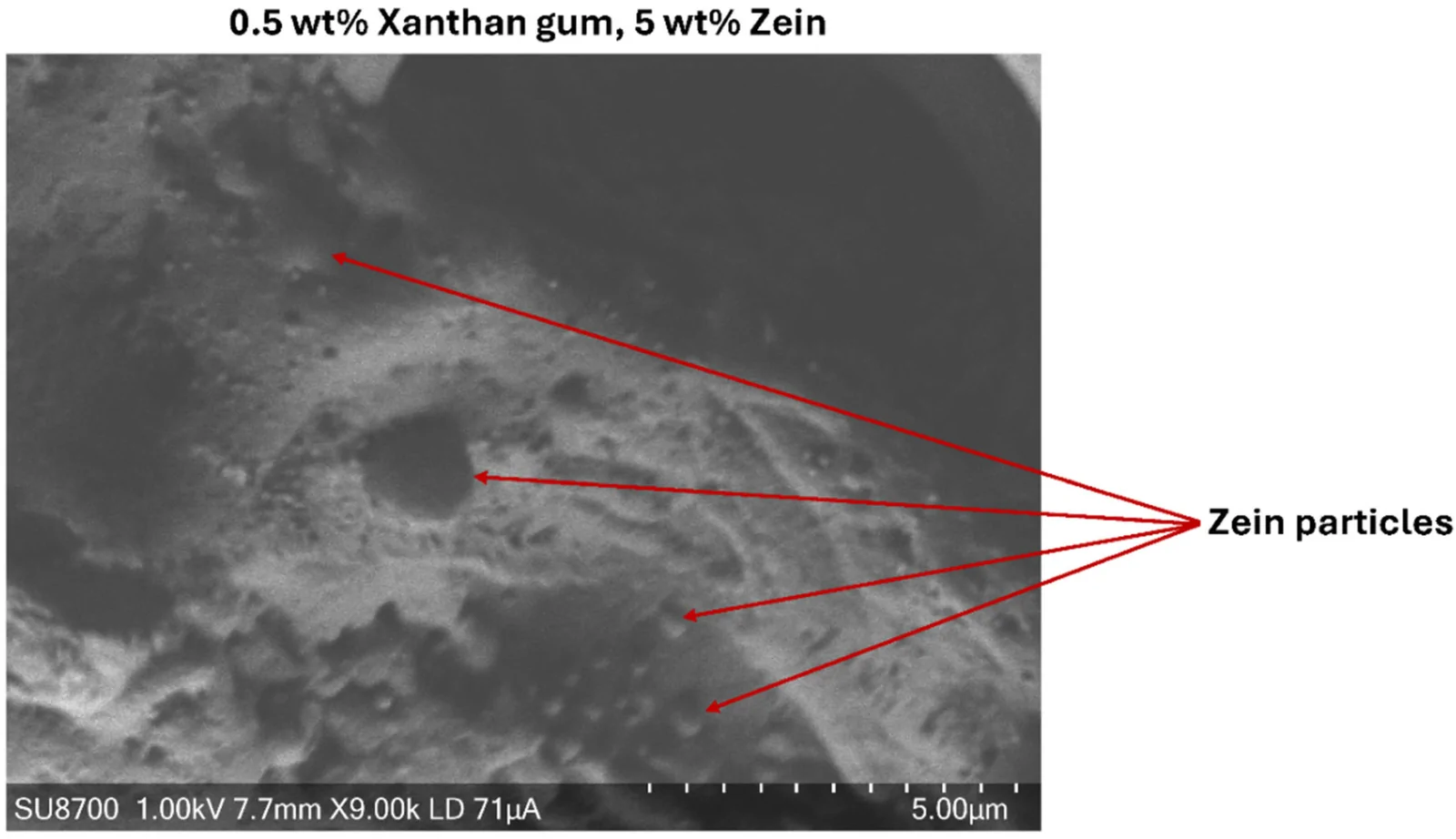
SEM image of zein–XG dispersion at zein concentration of 5 wt%.

It appears that, with the increase in the zein concentration, the clumping of zein nanoparticles takes place disrupting the entangled network of xanthan gum molecules, as shown schematically in Figure 23. The disruption of the entangled network of xanthan gum molecules decreases the consistency index and makes the dispersion less shear-thinning. Figure 24 shows the SEM image of zein–XG dispersion at 5 wt% zein. The lumps of zein nanoparticles can be seen in the image.

### 3.4. Surface Tension of Dispersions of Zein Nanoparticles in Xanthan Gum

**Figure 25:**
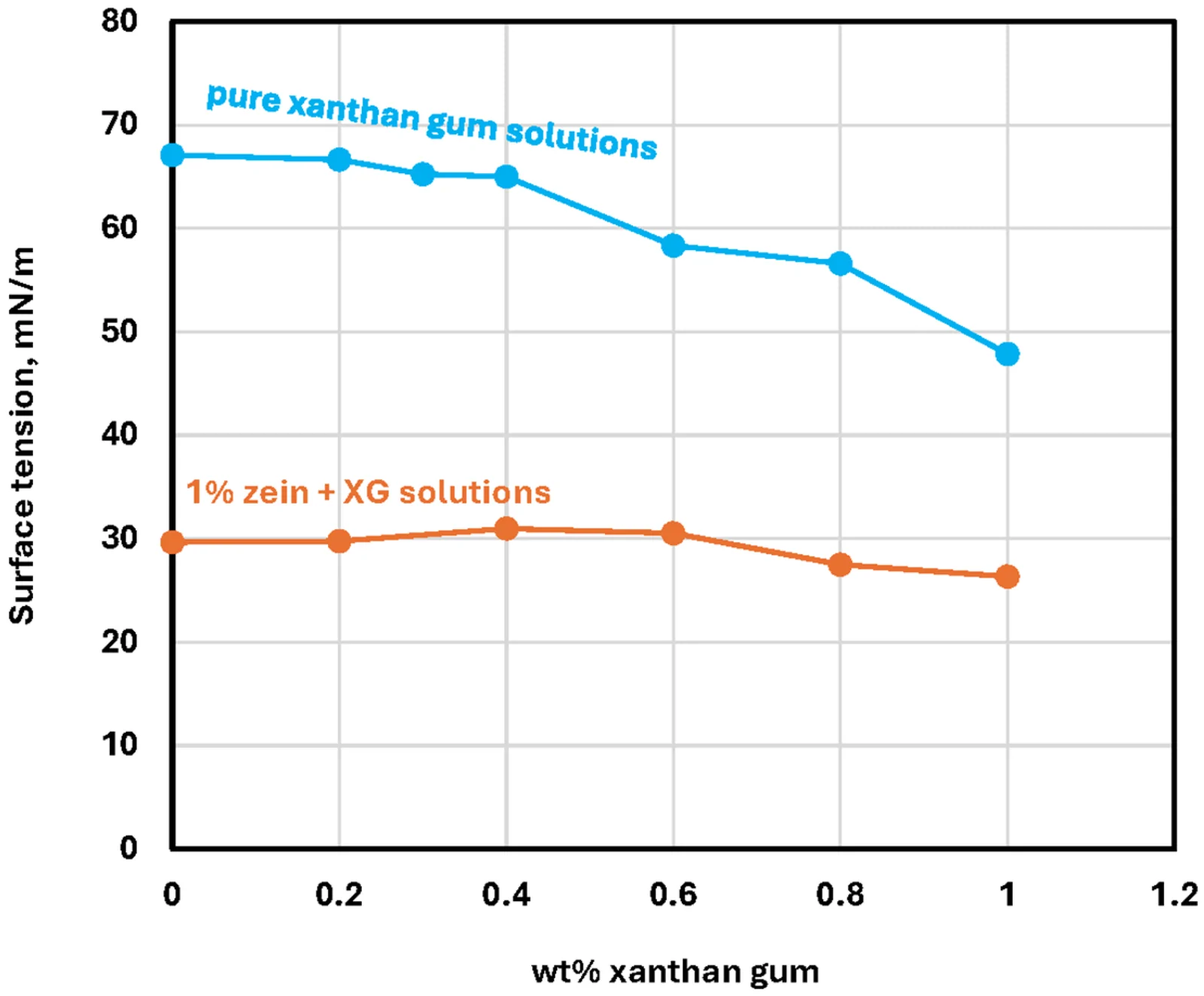
Surface tension of pure XG solutions (no zein) and zein–XG dispersions as function of XG concentration.

Figure 25 shows the variations of surface tension with the xanthan gum concentration of pure XG solutions and dispersions of zein nanoparticles in XG at a fixed zein concentration of 1 wt%. The pure XG solutions are surface-active in that the surface tension decreases with the addition of xanthan gum to deionized water. The surface tension continues to decrease with the increase in XG concentration. With the addition 1 wt% of zein nanoparticles to the zein–XG dispersions, the surface tension drops substantially, indicating that the zein nanoparticles are surface-active. Clearly, the surface-active zein-derived species (potentially including intact nanoparticles, dissociated protein, or protein–polysaccharide complexes) adsorb at the air/liquid interface, causing a sharp drop in the surface tension.

With the increase in XG concentration at a fixed zein nanoparticle concentration of 1 wt%, the surface tension remains constant up to XG concentration of 0.6 wt% and decreases with further increase in XG concentration.

**Figure 26:**
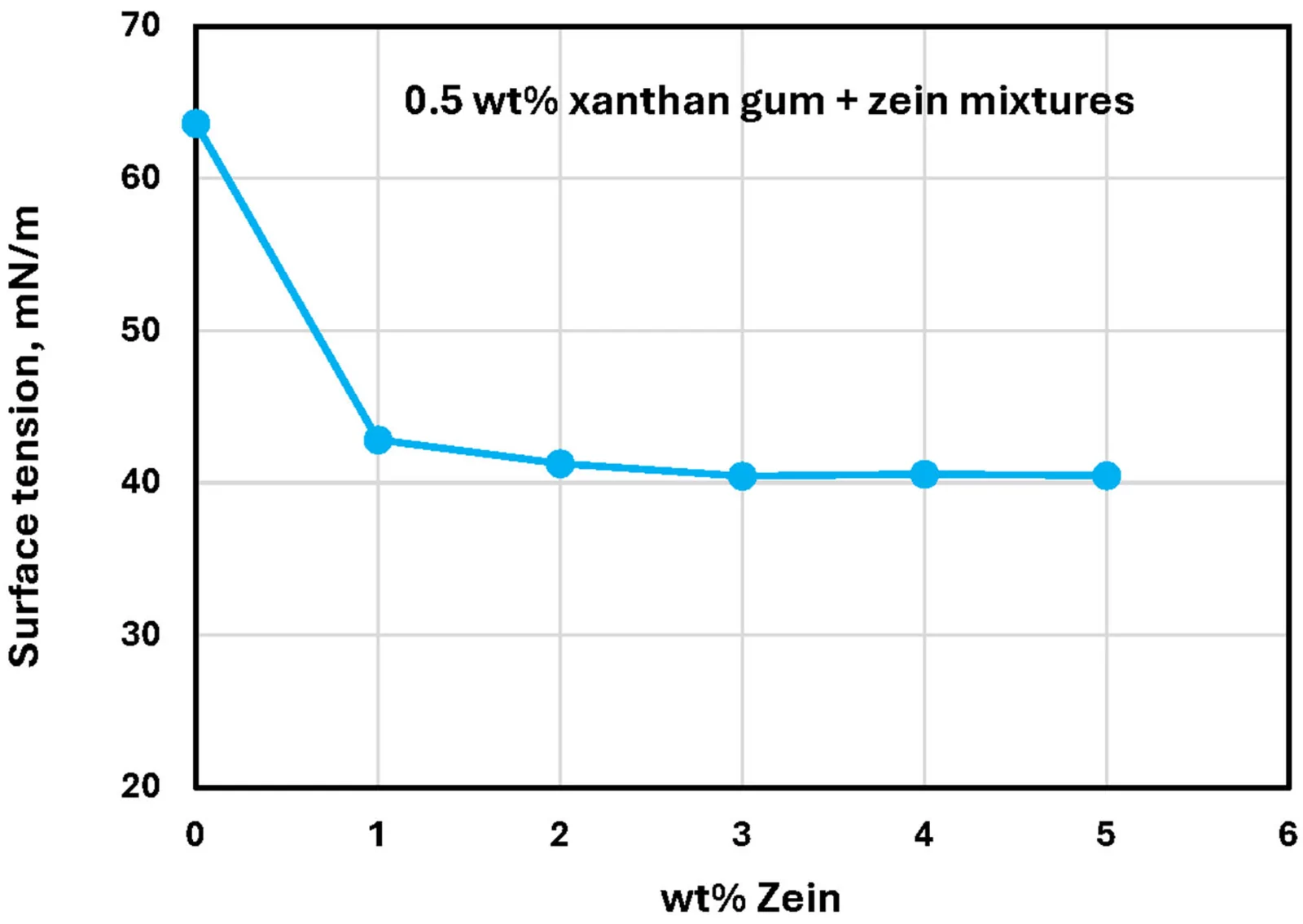
Effect of zein concentration on the surface tension of zein–XG dispersions at a fixed XG concentration of 0.5 wt%.

The effect of zein concentration on the surface tension of zein–XG dispersions at a fixed XG concentration of 0.5 wt% is shown in Figure 26. The surface tension drops sharply with the increase in zein concentration from 0 to 1 wt%. With further increase in zein concentration, the surface tension decreases slowly and levels off beyond 3 wt% zein. The decrease in surface tension is due to the increase in adsorption of zein nanoparticles at the air/liquid interface. At high zein concentrations, the interface becomes saturated with zein nanoparticles, and hence, the surface tension levels off. A similar behavior was observed in the case of zein–CMC dispersions.

## 4. Conclusions

The rheological behavior and surface-active properties of zein nanoparticle–polysaccharide composite systems fabricated via the anti-solvent precipitation method were investigated. The polysaccharides used were sodium carboxymethyl cellulose (CMC) and xanthan gum (XG). Based on the experimental results, the following conclusions can be made:

- Pure polymer solutions (CMC and XG) exhibit shear-thinning behavior over the concentration range investigated (0.2 to 1 wt%). XG solutions are much more viscous and shear-thinning compared with CMC solutions at the same polymer concentration.
- Zein nanoparticles are successfully fabricated in CMC and XG solutions using the anti-solvent precipitation method. The SEM images clearly show the presence of zein nanoparticles in CMC and XG solutions.
- Zein nanoparticle–CMC dispersions exhibit shear-thinning non-Newtonian behavior that could be described adequately using the power-law model. The dispersions of zein nanoparticle and CMC are much more viscous and shear-thinning in comparison with pure CMC solutions. With the increase in the zein concentration at a fixed CMC concentration of 0.5 wt%, the consistency rises substantially up to 4 wt% zein concentration and then drops sharply with further increase in zein concentration.
- Zein nanoparticle–XG dispersions also exhibit shear-thinning non-Newtonian behavior that could be described adequately using the power-law model. However, they behaved differently from zein nanoparticle–CMC dispersions. The dispersions of zein nanoparticle and XG are less viscous and shear-thinning in comparison with pure XG solutions over the XG concentration range of 0–0.7 wt%. At XG concentrations higher than 0.7 wt%, the zein nanoparticle-XG dispersions were more viscous and shear-thinning in comparison with pure XG solutions. Unlike zein nanoparticle–CMC dispersions, the consistency of zein nanoparticle-XG dispersions shows a persistent decline with the increase in zein concentration.
- The surface tension of polysaccharide solutions is strongly affected by the addition of zein nanoparticles. It decreases substantially with the addition of zein to CMC or XG solutions. The precise molecular identity of the interfacially active species (intact nanoparticles vs. dissociated/aggregated protein or protein–polysaccharide complexes) remains to be conclusively established.
- The transitions in rheological and surface-active properties of zein nanoparticle-polysaccharide dispersions are explained in terms of microstructure of the dispersions.

## 5. Recommendations for Future Work

This study involved some limitations that should be acknowledged. First, the final pH of the dispersions after ethanol evaporation and water back-dilution was estimated using pH indicator strips rather than precise electrode measurement, due to the limited volume of retained samples (Section 2.3). Second, residual ethanol content was not directly quantified; however, a mass-balance estimate based on gravimetric monitoring during evaporation, together with the surface tension of the polysaccharide-only control solutions, suggests that residual ethanol was reduced to a negligible level (Section 2.4). Third, the precise molecular identity of the species adsorbing at the air–liquid interface (intact zein nanoparticles, dissociated protein, or protein–polysaccharide complexes) could not be conclusively established from equilibrium surface tension data alone; dynamic (time-resolved) surface tension measurements and interfacial rheology would be needed to resolve this. Finally, both the rheological and surface tension measurements reported here were obtained via repeated technical measurements on a single preparation for each formulation, rather than from independently prepared replicate batches; this design establishes measurement reproducibility but not formulation-to-formulation (batch) reproducibility. Future studies incorporating independent replicate preparations, dynamic surface tension measurements, and direct quantification of residual solvent would help address these points. Future work should also cover dynamic (oscillatory) rheology to further probe the microstructure of zein protein nanoparticles-polysaccharides dispersions.

## Figures and Tables

**Table 1:** Radii and gap widths of co-axial cylinders of viscometers.

Viscometer Type	Radius of Inner Cylinder, $R_{i}$ (cm)	Radius of Outer Cylinder, $R_{o}$ (cm)	Length of Inner Cylinder (cm)	Gap-Width Between Cylinders (cm)
Fann viscometer (outer cylinder rotates, inner cylinder stationary)	1.72	1.84	3.8	0.12
Haake viscometer with MV I bob (inner cylinder rotates, outer cylinder stationary)	2.00	2.1	6.0	0.10

## Data Availability

The original contributions presented in the study are included in the article, further inquiries can be directed to the corresponding author.

## References

[1] Song J., Zhang L., Li X. (2020). Preparation and characterization of zein nanoparticles by anti-solvent precipitation method. J. Agric. Food Chem..

[2] Wang Y., Li H., Zhao Q., Chen L. (2024). Polysaccharide-mediated stabilization and functional improvement of zein-based colloidal systems. Food Chem..

[3] Patel A.R., Bouwens E.C.M., Velikov K.P. (2014). Preparation and characterization of zein nanoparticles. Food Hydrocoll..

[4] Benchabane A., Bekkour K. (2008). Rheological properties of carboxymethyl cellulose solutions. Colloid Polym. Sci..

[5] Behra J.S., Mattsson J., Cayre O.J., Robles E.S.J., Tang H., Hunter T.N. (2019). Characterization of sodium carboxymethyl cellulose aqueous solutions to support complex product formulation: A rheology and light scattering study. ACS Appl. Polym. Mater..

[6] Wang L., Xiang D., Li C., Zhang W., Bai X. (2021). Effects of lyophilization and low-temperature treatment on the properties and conformation of xanthan gum. Food Hydrocoll..

[7] Blok A.E., Bolhuis D.P., Kibbelaar H.V.M., Bonn D., Velikov K.P., Stieger M. (2021). Comparing rheological, tribological and sensory properties of microfibrillated cellulose dispersions and xanthan gum solutions. Food Hydrocoll..

[8] Zou T., Li Z., Percival S.S., Bonard S., Gu L. (2019). Fabrication, characterization, and cytotoxicity evaluation of zein nanoparticles for co-delivery of curcumin and quercetin. J. Agric. Food Chem..

[9] Zhong Q., Jin M. (2009). Zein nanoparticles produced by liquid–liquid dispersion. Food Hydrocoll..

[10] Wan Y., Liu C., Chen K., Zhang Y. (2023). Formation of zein–carboxymethyl cellulose nano-networks and their structural properties. Food Chem..

[11] Luo Y., Teng Z., Wang Q. (2011). Development of zein nanoparticles coated with carboxymethyl chitosan for encapsulation and controlled release. J. Agric. Food Chem..

[12] Santos J., Alcaide-González M.A., Trujillo-Cayado L.A., Alfaro-Rodríguez M.C. (2020). Development of food-grade Pickering emulsions stabilized by a biological macromolecule (xanthan gum) and zein. Int. J. Biol. Macromol..

[13] Liu Q., Li Y., Zhou Y., Chen L. (2022). Zein-whey protein isolate-carboxymethyl cellulose complex as carrier of apigenin via pH-driven method: Fabrication, characterization, stability, and in vitro release property. Food Chem..

[14] Giteru S.G., Oey I., Ali M.A., Johnson S.K., Fang Z. (2021). Rheological and structural properties of plant protein–polysaccharide composite systems: A review. Food Hydrocoll..

[15] Thoo Y.Y., Teo R.W., Ting A.S.Y. (2023). Effect of Zein Protein on Rheological and Hydrophobicity of Gellan Gum-based Food Packaging Film. Malays. J. Chem..

[16] Gagliardi A., Voci S., Salvatici M.C., Fresta M. (2020). Zein-based materials for biomedical and food applications: A review. Molecules.

[17] Li H., Zhao Q., Wang Y., Chen L. (2020). Interaction and rheological behavior of zein–polysaccharide composite systems. Int. J. Biol. Macromol..

[18] Joolaei Ahranjani P., Polo A., Di Cagno R., Ferrentino G. (2025). Comparative evaluation of stability and interfacial characteristics of Pickering emulsions co-stabilized by zein and hydrocolloids: Role of charge profile, emulsifying ability, and rheological properties. Food Res. Int..

[19] Sriprablom J., Suphantharika M. (2022). Influence of xanthan gum on properties and stability of oil-in-water Pickering emulsions stabilized by zein colloidal particles. J. Food Meas. Charact..

[20] Chen L., Xu W., Yang Z., McClements D.J., Peng X., Xu Z., Meng M., Zou Y., Chen G., Jin Z. (2024). Co-encapsulation of quercetin and resveratrol: Comparison in different layers of zein-carboxymethyl cellulose nanoparticles. Int. J. Biol. Macromol..

[21] Li M., Yu M. (2020). Development of a nanoparticle delivery system based on zein/polysaccharide complexes. J. Food Sci..

[22] Chen H., Muros-Cobos J.L., Holgado-Terriza J.A., Amirfazli A. (2017). Surface tension measurement with a smartphone using a pendant drop. Colloids Surf. A.

[23] Einstein A. (1906). Eine neue Bestimmung der Molekuldimension. Ann. Phys..

[24] Sayko R., Jacobs M., Dobrynin A.V. (2021). Quantifying Properties of Polysaccharide Solutions. ACS Polym. Au.

[25] Chen H., Wang Q., Rao Z., Lei X., Zhao J., Lei L., Ming J. (2023). The linear/nonlinear rheological behaviors of Pickering emulsion stabilized by Zein and Xanthan gum: Effect of interfacial assembly strategies. Food Hydrocoll..

[26] Bertsch P., Thoma A., Bergfreund J., Geue T., Fischer P. (2019). Transient measurement and structure analysis of protein-polysaccharide multilayers at fluid interfaces. Soft Matter.

